# Deep-Cavity Calix[4]naphth[4]arene Macrocycles: Synthesis, Conformational Features, and Solid-State Structures

**DOI:** 10.3390/molecules29174142

**Published:** 2024-08-31

**Authors:** Paolo Della Sala, Veronica Iuliano, Margherita De Rosa, Carmen Talotta, Rocco Del Regno, Placido Neri, Silvano Geremia, Neal Hickey, Carmine Gaeta

**Affiliations:** 1 Laboratory of Supramolecular Chemistry, Dipartimento di Chimica e Biologia “A. Zambelli”, Università degli Studi di Salerno, Via Giovanni Paolo II, 132, 84084 Fisciano, Italy; viuliano@unisa.it (V.I.); maderosa@unisa.it (M.D.R.); ctalotta@unisa.it (C.T.); rodelregno@unisa.it (R.D.R.); neri@unisa.it (P.N.); cgaeta@unisa.it (C.G.); 2 Centro di Eccellenza in Biocristallografia, Dipartimento di Scienze Chimiche e Farmaceutiche, Università di Trieste, Via L. Giorgieri 1, 34127 Trieste, Italy; nhickey@units.it

**Keywords:** deep-cavity hosts, hybrid macrocycles, fragment coupling synthesis

## Abstract

We recently introduced calix[*n*]naphth[*m*]arenes as a novel class of deep-cavity hybrid macrocycles constituted by phenol (n) and naphthalene (m) units. In this study, we report the synthesis, conformational analysis, spectroscopic properties, and solid-state structures of calix[4]naphth[4]arene (**C_4_N_4_**) and its permethylated analog (**C_4_N_4_-Me**), thereby expanding the calix[*n*]naphth[*m*]arene family. **C_4_N_4_** was synthesized through a 2 + 2 fragment coupling macrocyclization under acidic conditions, where the solvent played a crucial role in selectively forming the **C_4_N_4_** derivative. The X-ray structure of **C_4_N_4_** reveals a chair-like 1,2,3,4-alternate conformation characterized by two opposing 3/4-cone moieties stabilized by intramolecular hydrogen bonds. In contrast, the X-ray structure of **C_4_N_4_-Me** exhibits a 1,3,5,7-alternate conformation.

## 1. Introduction

Macrocycles play a crucial role in molecular recognition processes due to their capacity to mimic natural receptors. Among the various macrocycles studied in supramolecular chemistry, calixarenes, resorcinarenes, and pillararenes stand out for their remarkable supramolecular properties, attributed to their conformational flexibility and synthetic versatility [1,2,3,4].

In the past decade, considerable efforts have been dedicated to the synthesis and study of deep-cavity macrocycles, which can provide large contact surfaces with substrates [5,6,7,8]. Notably, saucerarenes [9], pagodarenes [10], naphthotubes [11], prismarenes [12], oxatubarenes [13], and calix[4]naphthalenes [14,15] have demonstrated intriguing molecular recognition abilities in both organic and aqueous media [16,17,18].

Many of these macrocycles are synthesized through one-pot condensations of monomeric aromatic units, such as *p-tert*-butylphenol, resorcinol, 1,4-dimethoxybenzene, substituted naphthalene, or anthracene, with paraformaldehyde or aliphatic aldehydes under acidic conditions. However, this synthetic approach does not easily allow for the synthesis of hybrid macrocycles composed of diverse building blocks, which could introduce novel and intriguing conformational and recognition properties.

**Figure 1:**
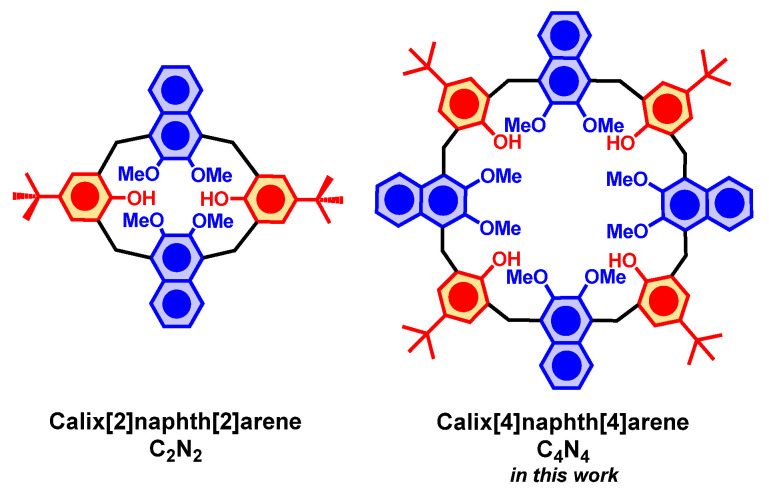
Chemical drawing of calix[n]naphth[m]arenes.

**Scheme 1:**
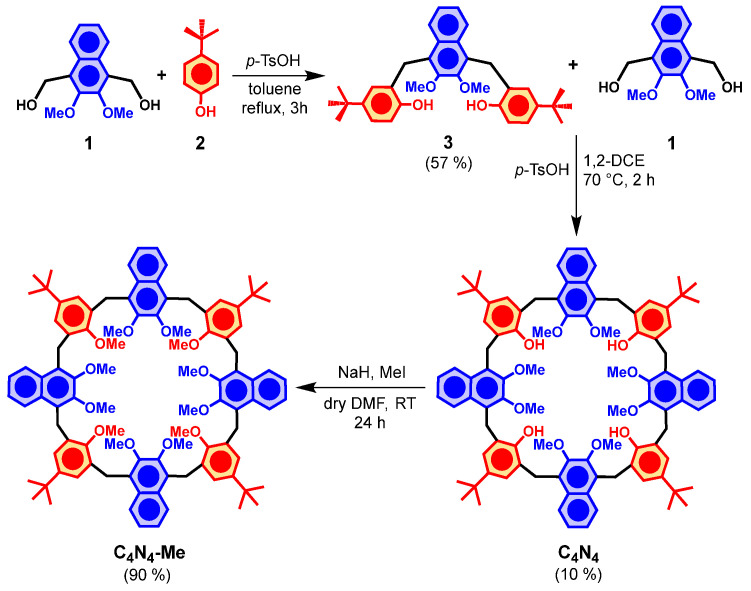
Fragment coupling synthesis of Calix[4]naphth[4]arene **C_4_N_4_**.

In 2020, our group reported the first example of a new class of hybrid macrocycles named calix[2]naphth[2]arene (**C_2_N_2_**, Figure 1), composed of alternating *p-tert*-butylphenol and 2,3-dimethoxynaphthalene units [19]. This macrocycle was synthesized using the fragment coupling synthesis (FCS, [20]) method outlined in Scheme 1, where derivatives **1** and **2** were reacted to yield derivative **3** with a 57% yield. Finally, **C_2_N_2_** in Figure 1 was obtained in a 26% yield through a 1 + 1 macrocyclization of **3** and **1** in *ortho*-dichlorobenzene (*o*-DCB). Calix[2]naphth[2]arene adopts a 1,2-alternate conformation both in the solid state and in solution, demonstrating molecular recognition abilities toward alkali metal cations. Furthermore, we observed that the stabilization of the alkali metal complexes was significantly driven by cation··π interactions between the cationic guests and the aromatic naphthalene walls. These intriguing conformational and supramolecular properties of **C_2_N_2_** prompted us to explore the synthesis and study of the larger homolog, calix[4]naphth[4]arene (**C_4_N_4_**, Figure 1).

## 2. Results and Discussion

### 2.1. Synthesis of Calix[4]naphth[4]arenes

The synthesis of calix[4]naphth[4]arene (**C_4_N_4_**) is outlined in Scheme 1. Derivative **3** [19] was obtained through the coupling reaction between **1** and *p-tert*-butylphenol (**2**), which serves as the synthetic precursor for both **C_2_N_2_** and **C_4_N_4_**.

To attempt the synthesis of the larger **C_4_N_4_**, we decided to vary the experimental conditions previously reported for the synthesis of **C_2_N_2_** [19], focusing our attention on the role of the solvent. It is well known that the solvent plays a crucial role in macrocyclization processes, acting as a templating agent [21,22]. For example, prism[5]arenes were obtained in the presence of 1,2-dichloroethane (1,2-DCE) [12,23], while a low yield was observed using *o*-DCB. Conversely, Chen and co-workers [24] demonstrated that *o*-DCB was effective in the synthesis of a triptycene-based calix[6]arene macrocycle, likely due to a solvent-template effect. Similarly, our group reported that *o*-DCB was also effective in the synthesis of resorcin[6]arenes [22]. In the cases of pillar[5]arene [25,26] and confused-prism[5]arene [12], it has been observed that the solvent acts as a template, facilitating and stabilizing the proximity of the two reactive ends of the linear oligomer. In both instances, halogenated solvents have proven to be the most effective templating agents. Solid-state analysis has shown the inclusion of the solvent molecules within the cavity of the formed macrocycles [12,25,26]. Consequently, the shape, size, and chemical nature of the solvent can significantly influence the reaction pathways, favoring the formation of a preferred macrocycle.

Prompted by these considerations, we attempted the macrocyclization of **1** and **3** in the presence of 1,2-DCE as the solvent and *p*-toluenesulfonic acid as the catalyst (0.5 eq), following the conditions previously reported for the synthesis of calix[2]naphth[2]arene [19]. Under these conditions, **C_4_N_4_** was isolated in a 5% yield after column chromatography, and the formation of calix[2]naphth[2]arene was not observed. The high-resolution FT-ICR MALDI mass spectrum revealed a molecular ion peak at *m*/*z* 1448.7565, corresponding to the molecular formula of **C_4_N_4_** (calculated *m*/*z* 1448.7522 for C_96_H_104_O_12_). The ^1^H NMR spectrum of **C_4_N_4_** (298 K in CD_2_Cl_2_, 400 MHz) showed a highly symmetrical, time-averaged C_4V_ structure, indicating conformational mobility of the macrocycle. This was confirmed by a singlet at 4.30 ppm, attributable to the methylene bridges (ArCH_2_Ar). Additionally, an AX system was observed at 8.07–7.19 ppm, corresponding to the naphthalene units. A singlet at 7.01 ppm was assigned to the *p-tert*-butylphenol moiety, while the OH groups produced a singlet at 7.29 ppm. Finally, the OMe signal was detected at 3.68 ppm.

With these results in hand, we explored the role of the solvent in the macrocyclization of compounds **1** and **3**, focusing on halogenated solvents such as chloroform, dichloromethane, and 1,1,2,2-tetrachloroethane (Table 1). Interestingly, this screening revealed that the most favorable selectivity for the **C_4_N_4_**/**C_2_N_2_** ratio was observed in 1,2-DCE (Table 1, entry 1).

Subsequently, we explored the influence of the acid catalyst during the macrocyclization in Scheme 1 to improve the yield of **C_4_N_4_**. When the reaction was performed with trifluoroacetic acid or triflic acid (15 or 30 equivalents, Table 2), no trace of the macrocycle was detected by TLC or mass spectrometry. However, when the amount of *p*-toluenesulfonic acid was increased to two or five equivalents, the yield of **C_4_N_4_** increased to 7% and 10%, respectively (Table 2).

Finally, calix[4]naphth[4]arene **C_4_N_4_** was exhaustively alkylated, using methyl iodide and sodium hydride as the base, in dry *N,N*-dimethylformamide for 24 h (Scheme 1). The dodecamethoxy-calixnaphtharene **C_4_N_4_-Me** was obtained with a 90% yield after purification by column chromatography. The high-resolution FT-ICR MALDI mass spectrum (Supporting Information) revealed a molecular ion peak at *m*/*z* 1504.8183, consistent with the molecular formula of **C_4_N_4_-Me** (calculated *m*/*z* 1504.8148 for C_100_H_112_O_12_). The signal pattern of the ^1^H NMR spectrum (CD_2_Cl_2_, 600 MHz) of **C_4_N_4_-Me** was consistent with a time-averaged C_4V_ structure of the macrocycle (vide infra). Specifically, the ^1^H NMR spectrum of **C_4_N_4_-Me** showed an AX system at 7.77 and 7.12 ppm attributable to the naphthalene rings and a singlet at 6.52 ppm attributable to the *p-tert*-butylphenol moiety. Additionally, the methylene bridges appeared as a singlet at 4.55 ppm, and two singlets for the OMe groups were observed at 4.06 and 3.92 ppm, corresponding to the phenol and naphthalene rings, respectively.

**Figure 2:**
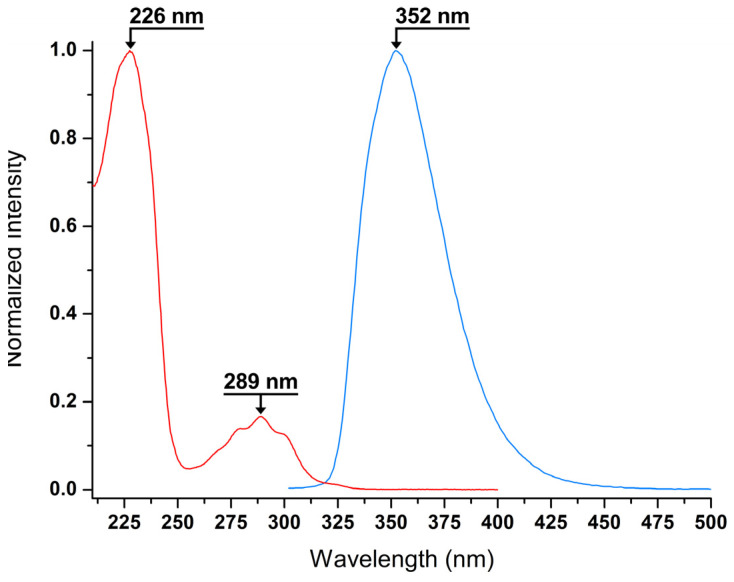
Normalized UV-vis absorption (red) and emission (light blue).spectra of **C_4_N_4_-Me** in *n*-hexane.

The UV-vis absorption spectrum of **C_4_N_4_-Me** (Figure 2) in *n*-hexane is characterized by an intense band peaking at 226 nm (ε = 22,100 M^−1^ cm^−1^) and a broad band at 289 nm (ε = 35,900 M^−1^ cm^−1^). The band at 289 nm exhibits an electronic transition rich in vibronic structure. The spectrum shows significant similarities to those of phenol-, anisole-, and 2,3-dimethoxynaphthalene-based derivatives. The fluorescence spectrum of **C_4_N_4_-Me** was then collected using excitation wavelengths of 226 nm or 289 nm. When the solutions of **C_4_N_4_-Me** were irradiated at 226 nm, an intense emission band was observed at 352 nm, with a fluorescence quantum yield of Φ = 0.69.

### 2.2. Conformations of Calix[4]naphth[4]arenes in Solution

**Figure 3:**
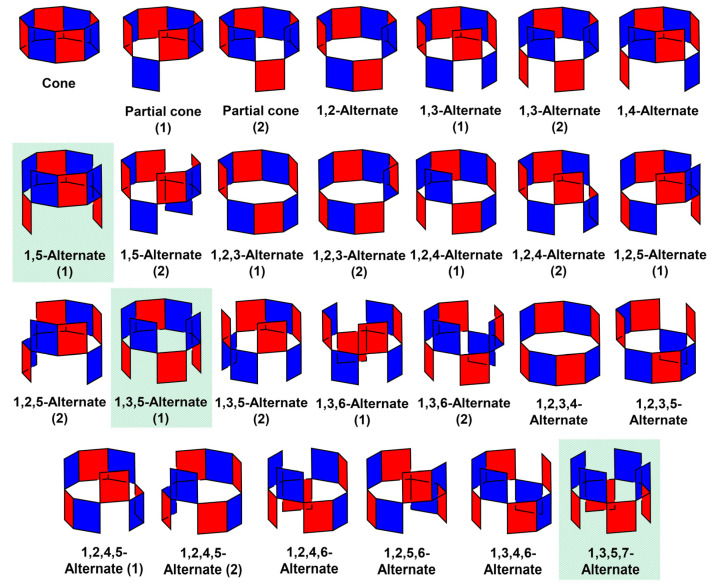
Schematization of the possible 27 discrete conformations of calix[4]naphth[4]arene, with phenol and naphthalene rings marked in red and blue, respectively.

Calix[2]naphth[2]arenes can adopt five conformations: cone, 1,2-alternate, 1,3-alternate, and two partial-cone forms, named partial-cone (1) and partial-cone (2) [19]. The conformational features of the larger calix[4]naphth[4]arene are markedly more complex than those of **C_2_N_2_** due to the larger number of monomeric units. The number of potential conformations increases drastically (27 in Figure 3) as a result of the various possible combinations of syn/anti arrangements of the aromatic rings and their inward/outward orientations. Additionally, the presence of two different aromatic units, phenol and naphthalene, results in an even higher number of “up-down” conformers compared to calix[8]arene, which exhibits only sixteen conformers [27].

**Figure 4:**
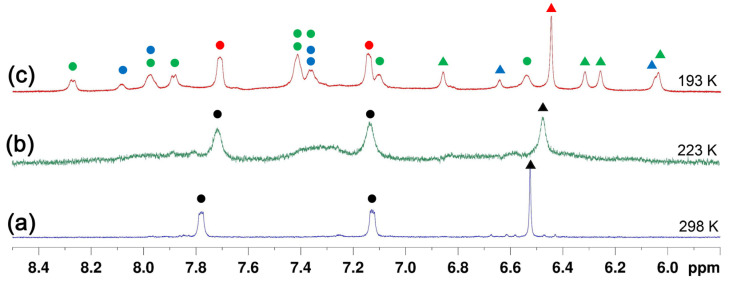
Aromatic portions of the ^1^H NMR spectra of **C_4_N_4_-Me** in CD_2_Cl_2_ at 600 MHz at (**a**) 298 K, (**b**) 223 K, and (**c**) 193 K. The signals of the aromatic hydrogen atoms of the naphthalene rings and phenol units are marked with circles and triangles, respectively. Blue, green, and red signals correspond to the 1,5-alternate (1), 1,3,5-alternate (1), and 1,3,5,7-alternate conformations, respectively.

**Figure 5:**
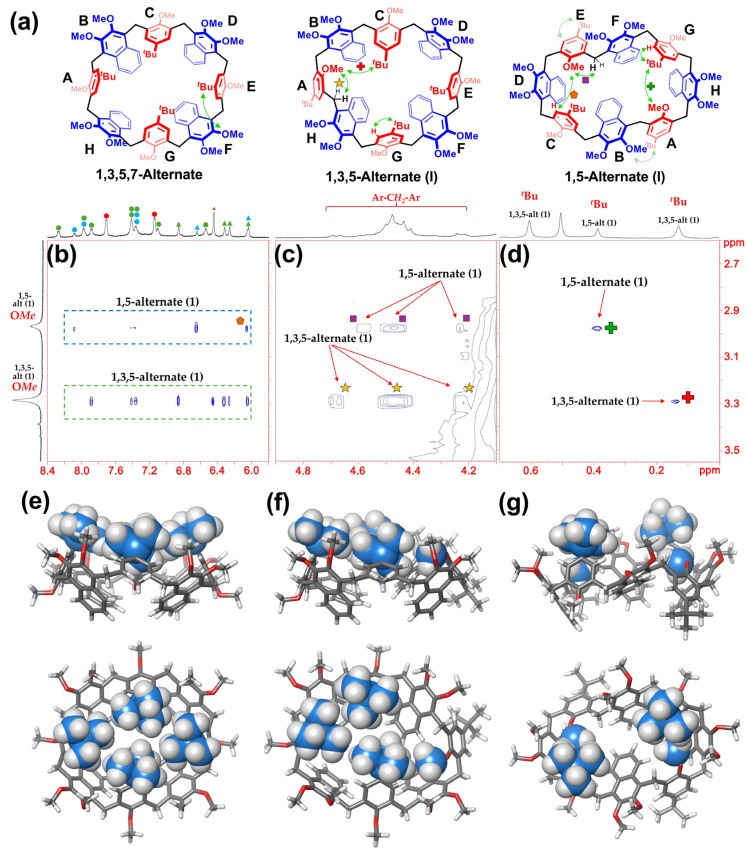
Chemical drawing of (**a**) 1,3,5,7- alternate, 1,3,5-alternate (1), and 1,5-alternate (1) conformations obtained by molecular dynamics calculation. (**b**–**d**) Significant portions of the 2D-NOESY spectrum of **C_4_N_4_-Me** (CD_2_Cl_2_, 600 MHz, 193 K). The signals of the aromatic hydrogen atoms of the naphthalene rings and phenol units are marked in “b” with circles and triangles, respectively. Blue, green, and red signals are attributable to 1,5-alternate (1), 1,3,5-alternate (1), and 1,3,5,7-alternate conformations, respectively. (**e**–**g**) Self-filling of the cavities of **C_4_N_4_-Me** in 1,3,5,7- alternate, 1,3,5-alternate (1), and (**c**) 1,5-alternate (1) conformations, respectively, obtained by molecular dynamics calculation in top and side views.

Dynamic NMR studies (CD_2_Cl_2_, 600 MHz, Figure 4 and Supporting Information) were performed on **C_4_N_4_** and **C_4_N_4_-Me** derivatives to investigate their conformational properties in solution. As the temperature was lowered, the ^1^H NMR spectrum of **C_4_N_4_** showed a broadening of the signals, indicating conformational mobility of the macrocycle (SI). Even when the temperature was reduced to 193 K, the ^1^H NMR spectrum of **C_4_N_4_** remained broad, preventing the acquisition of detailed conformational information. The variable-temperature (VT) NMR studies performed on **C_4_N_4_-Me** revealed a coalescence temperature of 223 K (Figure 4b). Upon lowering the temperature to 193 K, sharp signals appeared (Figure 4c and Supporting Information) in the ^1^H NMR spectrum of **C_4_N_4_-Me**, attributable to conformations frozen within the NMR time scale. In silico calculations performed with Yasara software [28,29] suggested that the most stable conformations are the 1,3,5,7-alternate, 1,3,5-alternate (1), and 1,5-alternate (1) (vide infra, Figure 5).

*1,3,5,7-Alternate conformation*. Upon close inspection of the 1D and 2D NMR spectra (Figure 4c, red signals and Supporting Information), we assigned the naphthalene aromatic signals at 7.71 and 7.14 ppm and the anisole aromatic singlet at 6.45 ppm to the 1,3,5,7-alternate conformation (red signals in Figure 4c). The COSY spectrum (Figure S12) indicated a long-range coupling among these aromatic signals and the ArCH_2_Ar methylene bridge at 4.43 ppm. The energy-minimized structure of the 1,3,5,7-alternate conformation of **C_4_N_4_-Me** (Figure 5e) shows a symmetrical folded structure with *tert*-butyl groups and aromatic rings tilted inwards to fill the cavity (Figure 5e). The interproton distance of 3.9 Å between the aromatic hydrogen of the anisole rings (singlet at 6.45 ppm) and the aromatic hydrogen atom at position 6 of the naphthalene units (signal at 7.71 ppm) was determined through NOESY experiments conducted in CD_2_Cl_2_ at 193 K, following an established procedure [30,31]. In detail, following the method previously reported by Klochkov [30,31], a series of 2D NOESY NMR experiments in CD_2_Cl_2_ at 193 K with various mixing times were conducted in a regime of fast spectrum recording mode to measure the interproton distances. This measurement corroborates the folded structure predicted by computational methods and is further validated by X-ray crystallography.

The *1,3,5-alternate (1)* conformation is characterized by four AX systems at 8.27/7.40, 7.98/7.41, 7.88/7.34, and 7.11/6.54 ppm and four signals at 6.86, 6.32, 6.26, and 6.04 ppm attributable to the naphthalene and phenol units, respectively (Figure 4c, green signals). Two AB systems emerged in the COSY spectrum, attributable to the methylene bridge at 4.68/4.22 ppm (*J* = 16.4 Hz) and 4.50/4.41 ppm (*J* = 15.5 Hz), with two overlapping signals between 4.50 and 4.41 ppm. The 2D NOESY spectrum revealed the presence of diagnostic dipolar couplings between the methoxy signals (A in Figure 5a,d) at 3.29 ppm and the signals of the naphthalene rings. Moreover, the 2D NOESY experiment evidenced the presence of diagnostic dipolar couplings between the OMe singlet (A), tilted inside the cavity, and the AX system of the methylene bridge at 4.68/4.22 ppm (Figure 5c, marked with a star), as well as between the OMe singlet (A) and the *tert*-butyl singlet (C, Figure 5d marked with a red cross). This result aligns with the energy-minimized structure in Figure 5f (Supporting Information). Analogously, the 1,3,5-alternate (1) conformation was confirmed using a fast NOESY experiment conducted in CD_2_Cl_2_ at 193 K, which enables the measurement of proton distances in solution [30,31]. Specifically, a distance of 3.3 Å (see Table S4) was measured between the OMe group, positioned within the cavity (ring A in Figure 5a), and the *tert*-butyl group of ring C (see Figure 5a). Furthermore, the distance between the same OMe group and the aromatic hydrogen at position 6 of the naphthalene units was found to be 3.5 Å (Table S4). Additionally, the cross peaks corresponding to the two protons at position 6 of the distal naphthalene rings allowed us to estimate an average distance of 2.6 Å (Table S4), which aligns well with the structures obtained through molecular dynamics simulations.

Finally, the *1,5-alternate (1)* conformation is characterized by two AX systems at 8.08/7.37 and 7.97/7.36 ppm for the naphthalene ring and two broad singlets at 6.64 and 6.03 ppm for the phenol moieties. The singlet of the methoxy groups (E≡A) of the tilted *p-tert*-butylanisole units was shielded at 2.96 ppm.

Dipolar couplings between the singlet of the OMe group (E in Figure 5) and the naphthalene rings were observed in the 2D NOESY spectrum (Figure 5b). Notably, a dipolar coupling was observed between the shielded aromatic signal of the phenol ring marked with C and the OMe group of the phenol ring marked with E (marked with an orange pentagon in Figure 5b). Furthermore, the shielded OMe singlet exhibited dipolar couplings with the AX system of the methylene bridge at 4.58/4.21 ppm and the *p-tert*-butyl group (marked, respectively, with a purple square and a green cross in Figure 4d and Figure 5c). Furthermore, NOESY experiments enabled us to measure an interproton distance of 2.7 Å between the OMe group, positioned within the cavity (ring A in Figure 5a), and the *tert*-butyl group. Additionally, we observed a distance of 3.6 Å between the *tert*-butyl group and the aromatic hydrogen at position 6 of the naphthalene ring (Table S5). These measurements support the folded structure predicted by computational methods.

Integration of the ^1^H NMR signals for the 1,3,5-alternate (1), 1,3,5,7-alternate, and 1,5-alternate (1) conformers indicated a ratio of 47/38/15. Computational investigations performed with Yasara software [28,29] are in good agreement with these results, showing a 55/41/4 ratio among the conformers with ΔE of 0.12 and 1.00 kcal∙mol^−1^ relative to the most stable conformation.

### 2.3. Crystallographic X-ray Structure Determination of Calix[4]naphtharenes

**Figure 6:**
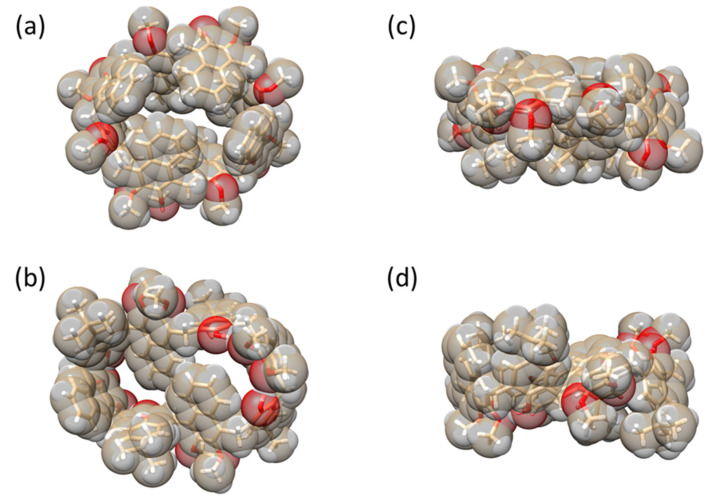
Different views of the X-ray structures of C_4_N_4_-Me (top view (**a**) and side view (**c**)) and C_4_N_4_ (top view (**b**) and side view (**d**)) The molecules are represented as capped stick models within their van der Waals surfaces, which are made transparent to aid visualization. For **C_4_N_4_-Me**, the side with the naphthalene groups is shown in the top view. The two sides are equivalent for **C_4_N_4_**.

**Figure 7:**
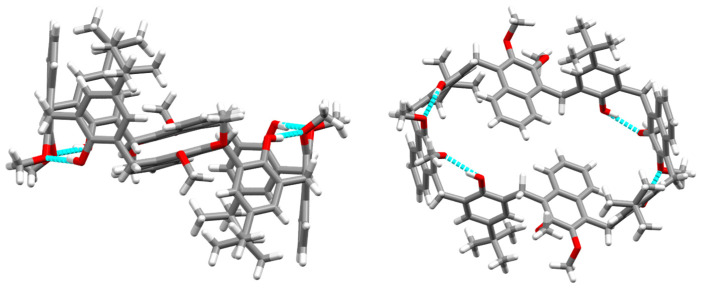
Side view (**left**) and top view (**right**) of the X-ray structure of **C_4_N_4_** in the chair-like conformation; the O−H···O hydrogen bonds are also represented.

**Figure 8:**
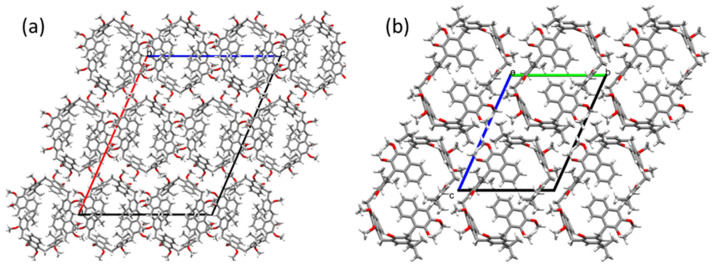
Crystal packing of **C_4_N_4_-Me**, as viewed along the b-axis (**a**), and **C_4_N_4_**, as viewed along the a-axis (**b**). Atoms are drawn with CPK colors.

Single crystals suitable for X-ray diffraction (XRD) analysis were obtained by the slow evaporation of hexane/dichloromethane solutions containing **C_4_N_4_-Me** or **C_4_N_4_** (Figure 6, Figure 7 and Figure 8 and Supporting Information). **C_4_N_4_-Me** exhibits the 1,3,5,7-alternate conformation (Figure 6), as described in Figure 3 and Figure 5. In this conformation, all four *tert*-butyl groups are positioned on one face of the macrocycle, while all four naphthalene groups are located on the opposite face, with all these moieties tilted towards the center of the cavity.

Dihedral canting angles between the mean planes of each of the eight calixarene/naphthalene aromatic rings and the mean plane defined by the methylene bridges are reported in Table S3. Since all the aromatic rings are tilted inward for **C_4_N_4_-Me**, all angles are less than 90° (angles greater than 90° would indicate a ring tilted outward from the center of its side of the macrocycle). Furthermore, due to the molecule’s C_2_ symmetry, there are only four unique angles, each of which is repeated twice. Two of the naphthalene groups lie quite flat in the cavity compared to the other two (26.77° vs. 64.91°). The differences between the pairs of canting angles of the *p-tert*-butyl-anisole rings are smaller (49.74° vs. 72.47°). **C_4_N_4_**, on the other hand, exhibits a chair-like [32] conformation corresponding to the 1,2,3,4-alternate conformation (Figure 6 and Figure 7). In this conformation, four of the aromatic rings (two naphthalene rings and two calixarene rings) lie on one side of the mean plane of the methylene bridges, while four equivalent rings (related by inversion) lie on the other side, making the two sides of the macrocycle identical. For each side, one of the naphthalene rings is tilted inwards, while the other three are tilted outwards (Figure 6 and Figure 7). The chair-like conformation of **C_4_N_4_** is characterized by two opposite 3/4-cone segments, reminiscent of the chair-like conformation previously observed in *p-tert*-butylcalix[8]arene [32]. Similar to **C_4_N_4_**-**Me**, the symmetry of the molecule (Ci) results in only four unique angles, each of which is repeated twice (Table S3).

Calculations indicate that this conformer is not among the more stable conformations for **C_4_N_4_**-**Me**. However, in the case of **C_4_N_4_**, there are two pairs of O-H···O hydrogen bonds (O-O distances of 2.68 to 2.70 Å), related by the inversion center at the center of the macrocycle (Figure 7 and Figure S16), which contribute to the stabilization of this conformation. These results suggest that for **C_4_N_4_**, the 1,2,3,4-alternate conformation is primarily stabilized by hydrogen bonding due to the presence of hydroxyl (OH) groups. When these OH groups undergo exhaustive alkylation in **C_4_N_4_**-**Me**, the hydrogen bonding interactions are lost. This loss is significant as it affects the molecule conformation. In the alkylated derivative, **C_4_N_4_**-**Me**, the absence of hydrogen bonds disrupts the stabilizing forces that favored the 1,2,3,4-alternate conformation of **C_4_N_4_**. Consequently, **C_4_N_4_**-**Me** adopts the 1,3,5,7-alternate conformation, which minimizes steric hindrance and allows for optimal packing. This folded 1,3,5,7-alternate conformation is crucial because it maximizes van der Waals interactions by enabling closer proximity of atoms or functional groups, enhancing stability despite the lack of hydrogen bonding. Thus, the conformations of **C_4_N_4_**-**Me** and **C_4_N_4_** illustrate a delicate balance between different molecular interactions that govern their structural properties.

The crystal packing of **C_4_N_4_**-**Me** and **C_4_N_4_** is shown in Figure 8. For **C_4_N_4_**-**Me**, the central cavities of the macrocycle are aligned along the b-axis. However, the eight aromatic rings, all tilted inwards as described above, effectively close off the cavity above and below. In the case of **C_4_N_4_**, the macrocycle cavities are aligned along the a-axis. The two inward-oriented naphthalene rings lie quite flat with respect to both the mean plane of the methylene groups (dihedral angles = 30.21°) and the bc plane. This configuration divides each cavity into two opposing sections resembling two ¾ partial cones, with the fourth ring being flat rather than inverted. Each of these sections forms isolated cavities with the corresponding sections of adjacent molecules stacked along the *a*-axis.

All chemical reagents were purchased from TCI, Fluorochem, and Merck, with no additional purification. Molecular sieves were activated at 200 °C in a vacuum over 48 h. All reaction solvents were dried by activated 3 Å molecular sieves [33]. Reaction temperatures were measured externally and were monitored by Merck TLC silica gel plates (0.25 mm) and visualized by UV light at 254 nm or by spraying with H_2_SO_4_-Ce(SO_4_)_2_. NMR spectra were acquired using Bruker Avance-600 [600 (^1^H) and 150 MHz (^13^C)] and Avance-400 [400 (^1^H) and 100 MHz (^13^C)] spectrometers. Chemical shifts are referenced to the residual solvent peak [34]. Standard pulse programs provided by the manufacturer were used for 2D COSY (cosygpqf), 2D HSQC (hsqcedetgpsisp2.2), and 2D NOESY (noesygpphpp) experiments. Structural assignments were made with additional information from gCOSY and gHSQC experiments. HR MALDI mass spectra were recorded on a Bruker Solaris XR Fourier transform ion cyclotron resonance (FT-ICR) mass spectrometer equipped with a 7 T refrigerated actively shielded superconducting magnet. Both samples were ionized using the MALDI ion source in positive mode, and 16 laser shots were used for each scan. The mass spectra were calibrated externally using a linear calibration. All samples were prepared by mixing 10 μL of analyte in dichloromethane (1 mg/mL) with 10 μL of a solution of 2,5-DHB in acetone (10 mg/mL).

Molecular dynamics calculations and energy minimization were performed on Intel Xeon GOLD 5118 processors and Intel Core i7-6700HQ.

The trimer **3** was synthesized according to the literature procedures [19], and ^1^H and ^13^C NMR spectra are in accordance with those reported in the literature.

## 3. Materials and Methods

### 3.1. Synthesis of C_4_N_4_

To a solution of **1** (53.0 mg, 0.21 mmol) and **3** (110.0 mg, 0.21 mmol) in 1,2-DCE (96 mL), molecular sieves (3.0 g, 3 Å) and *p*-toluensulfonic acid (200.0 mg, 1.05 mmol) were added. The mixture was stirred at 70 °C for 2 h under a nitrogen atmosphere. The solution was filtered through a Celite^®^ plug after the addition of 25 mL of water, and the organic phases were extracted and the solvent removed under reduced pressure. The mixture was separated by a chromatographic column on silica gel (Hexane/CH_2_Cl_2_/Et_2_O = 55/40/5, *v*/*v*/*v*) to give pure **C_4_N_4_** as a white powder (0.030 g, 10%).

**^1^H NMR** (400 MHz, CD_2_Cl_2_, 298 K): δ 8.06 (m, 8H, Ar-H), 7.29 (s, 4 H, Ar-OH), 7.19 (m, 8 H, Ar-H), 7.01 (s, 8 H, Ar-H), 4.30 (s, 16 H, Ar-CH_2_-Ar), 3.68 (s, 24 H, Ar-OCH_3_), 1.03 (s, 36 H, Ar-C(CH_3_)_3_).

**^13^C-NMR {^1^H}** (100 MHz, CD_2_Cl_2_, 298 K): δ (ppm): 150.4, 149.5, 142.3, 130.9, 128.2, 126.4, 126.0, 125.4, 125.1, 61.3, 34.0, 31.5, 27.0.

**HRMS** (FT-ICR-MALDI): *m*/*z* [M]^+^ calcd for C_96_H_104_O_12_: 1448.7534; found: 1448.7565.

**Mp**: >300 °C dec.

### 3.2. Synthesis of C_4_N_4_-Me

To a solution of **C_4_N_4_** (40.0 mg, 27.6 µmol) in dry DMF (10 mL), NaH (22.0 mg, 60% dispersion in mineral oil, 0.55 mmol) was added under a nitrogen atmosphere at 0 °C. The mixture was stirred for 15 min at room temperature. Then, methyl iodide (470.0 mg, 3.31 mmol) was added, and the resulting solution was stirred for 24 h at room temperature. After, 10 mL of 1 M solution of HCl was added. The mixture was extracted with CH_2_Cl_2_ (2 × 10 mL), and the organic layer was dried over Na_2_SO_4_, filtered, and concentrated under reduced pressure. The crude was purified through a chromatographic column on silica gel with CHCl_3_ to give pure **C_4_N_4_-Me** as a white powder (0.037 g, 90%).

**^1^H NMR** (600 MHz, CD_2_Cl_2_, 298 K): δ 7.78 (m, 8H, Ar-H), 7.12 (m, 8H, Ar-H), 6.52 (s, 8H, Ar-H), 4.55 (s, 16H, Ar-CH_2_-Ar), 4.06 (s, 12H, Ar-OCH_3_), 3.92 (s, 24H, Ar-OCH_3_), 0.50 (s, 36H, Ar-C(CH_3_)_3_).

**^13^C-NMR {^1^H}** (100 MHz, CD_2_Cl_2_, 298 K): δ (ppm): 153.0, 150.6, 146.7, 133.4, 130.8, 128.6, 125.2, 125.1, 124.4, 61.8, 61.3, 31.2, 24.8.

**HRMS** (FT-ICR-MALDI): *m*/*z* [M]^+^ calcd for C_100_H_112_O_12_: 1505,8182; found: 1505.8183.

**Mp**: >300 °C dec.

### 3.3. Computational Study

Conformational studies have been performed using the Yasara software package (version 22.9.24) [24,25] in a cubic box (with periodic boundaries) of dichloromethane (35 × 35 × 35 Å) and AMBER14 force field in NTV ensemble at 193 K for 10 ns. All conformers were manually prepared for the molecular dynamic simulations. The systems were then energy minimized using first the steepest descent minimization and then by a simulated annealing minimization until convergence. Energy-minimized structures obtained through dynamic simulation were used as a starting point for energy minimization using Yasara software and NOVA force field [28,29], converging as soon as the energy improves by less than 0.01 kcal/mol.

### 3.4. Single Crystal X-ray Diffraction

Single crystals suitable for X-ray diffraction (XRD) analysis were obtained through the slow evaporation of hexane/dichloromethane solutions containing **C_4_N_4_-Me** and **C_4_N_4_**. Data collection was performed at the Macromolecular Crystallography XRD1 beamline of the Elettra Synchrotron in Trieste, Italy. The rotating crystal method was utilized in conjunction with a Dectris Pilatus 2M area detector. The single crystals analyzed were dipped in paratone cryoprotectant, mounted on a nylon loop, and flash-frozen under a nitrogen vapor stream at 100 K.

Diffraction data were indexed and integrated using the XDS software package [35], while scaling was performed with XSCALE [36]. The structures were solved using the SHELXT program [37] and refined with the SHELXL-19/3 program [38] by full-matrix least-squares methods on F2, operating through the WinGX GUI [39]. Non-hydrogen atoms were refined anisotropically. Hydrogen atoms were positioned at calculated locations and refined using the riding model. Crystallographic data and refinement details are presented in the ESI. The X-ray crystallographic coordinates of the structures reported in this study were deposited at the Cambridge Crystallographic Data Centre (CCDC) under the deposition numbers 2373754 and 2373755. These data can be obtained free of charge from the CCDC at www.ccdc.cam.ac.uk/data_request/cif, accessed on 22 August 2024.

### 3.5. UV-Vis and Fluorescence Caracterization

Uv-vis spectra were registered in a 1 cm Quartz cuvette using hexane as a solvent on a Cary 50 UV-vis spectrophotometer, Varian. The extinction coefficient of **C_4_N_4_-Me** was determined by calculating the slope of the Lambert–Beer plot.

Fluorescence spectra were recorded using a 1 cm Quartz cuvette and hexane as a solvent on a Cary Eclipse Spectrophotometer (Varian, Australia).

The quantum yield of **C_4_N_4_-Me** was measured at room temperature after irradiation at 289 nm (see Supporting Information) using anthracene in ethanol as the external standard. The fluorescence of **C_4_N_4_-Me** was integrated from 330 to 500 nm, while the fluorescence of anthracene was integrated from 300 to 480 nm.

### 3.6. 2D NOESY Experiments

The determination of interproton distances using a fast NOESY experiment was carried out following a procedure previously reported by Klochkov and coworkers [30,31]. For NOESY experiments, a τ_mix_ = 0.1 s was selected (D1 = 1 s). When a 2D spectrum was processed, the Gaussian line shape and a digital resolution of 2K × 2K were used for digital filtration, with the number of scans being 32. The mixing time τ_mix_ ranged from 0.05 to 0.4 s in agreement with a procedure previously reported [40].

## 4. Conclusions

In conclusion, we present a new member of the calix[n]naphth[m]arene family, calix[4]naphth[4]arene (**C_4_N_4_**), and its permethylated analogue (**C_4_N_4_**-**Me**). We conducted a comprehensive investigation into the selective synthesis of this compound, exploring various solvents and acids, and identified 1,2-dichloroethane and *p-*toluenesulfonic acid as the optimal choices. Conformational studies utilizing low-temperature NMR experiments and computational methods revealed the presence of three conformers for **C_4_N_4_**-**Me**: 1,3,5,7-alternate, 1,3,5-alternate (1), and 1,5-alternate (1). The single crystal X-ray structural analysis reveals a chair-like conformation for **C_4_N_4_** around an inversion center located at the center of the macrocycle. This conformation is stabilized by four intramolecular hydrogen bonds and is characterized by two equivalent ¾ cone moieties created by two inward-orientated naphthalene groups that effectively split the cavity in two. The six other aromatic rings are orientated outwards from the center of the cavity. Each of these ¾ cone moieties forms an isolated, closed cavity in combination with an adjacent molecule stacked along the a-axis. The X-ray structure of **C_4_N_4_**-**Me** exhibits a 1,3,5,7-alternate conformation around a two-fold axis, which passes through the center of the cavity. All of the phenol and naphthalene aromatic rings are inward-orientated, which seals off the macrocycle cavity.

## Figures and Tables

**Table 1:** Yields of **C_4_N_4_** and **C_2_N_2_** in the presence different solvents.

Solvent	Temperature [°C]	C_2_N_2_ [%]	C_4_N_4_ [%]
1,2-DCE	70	-	5
*o*-DCB	100	26	-
CH_2_Cl_2_	40	-	-
CHCl_3_	60	trace	4
1,1,2,2-TCE	100	20	6

**Table 2:** Yields of **C_4_N_4_** and **C_2_N_2_** in the presence of different acids in 1,2-DCE at 70 °C.

Acid	Equivalents	C_2_N_2_ [%]	C_4_N_4_ [%]
*p*-Toluenesulfonic acid	0.5	-	3
*p*-Toluenesulfonic acid	1.0	-	6
*p*-Toluenesulfonic acid	2.0	-	7
***p*-Toluenesulfonic acid**	**5.0**	**-**	**10**
Trifluoroacetic acid	15	-	-
Trifluoroacetic acid	30	-	-
Trifluoromethanesulfonic acid	15	-	-

## Data Availability

The data presented in this study are available upon request from the corresponding authors.

## Supplementary Materials

The following supporting information can be downloaded at: https://www.mdpi.com/article/10.3390/molecules29174142/s1. Figure S1: ^1^H NMR spectrum of **C_4_N_4_** (CD_2_Cl_2_, 400 MHz, 298 K); Figure S2: ^13^C NMR {1H} spectrum of **C_4_N_4_** (CD_2_Cl_2_, 100 MHz, 298 K); Figure S3: 2D-HSQC spectrum of **C_4_N_4_** (CD_2_Cl_2_, 400 MHz, 298 K); Figure S4: Significant portion of the HR MALDI FT-ICR mass spectrum of **C_4_N_4_**; Figure S5: ^1^H NMR spectrum of **C_4_N_4_** (CD_2_Cl_2_, 600 MHz, 183 K); Figure S6: ^1^H NMR spectrum of **C_4_N_4_-Me** (CD_2_Cl_2_, 600 MHz, 298 K); Figure S7: ^13^C NMR {1H} spectrum of **C_4_N_4_-Me** (CD_2_Cl_2_, 100 MHz, 298 K); Figure S8: 2D-DQF COSY spectrum of **C_4_N_4_-Me** (CD_2_Cl_2_, 400 MHz, 298 K); Figure S9: 2D-HSQC spectrum of **C_4_N_4_-Me** (CD_2_Cl_2_, 400 MHz, 298 K); Figure S10: Significant portion of the HR MALDI FT-ICR mass spectrum of **C_4_N_4_-Me**; Figure S11: ^1^H NMR spectra of **C_4_N_4_-Me** (CD_2_Cl_2_, 600 MHz) at (from bottom to top): 298, 283, 273, 263, 253, 243, 233, 223, 213, 203, 193 and 183 K; Figure S12: Portions of 2D-DQF COSY spectrum of **C_4_N_4_-Me** (CD_2_Cl_2_, 600 MHz, 193 K). The signals of the aromatic hydrogen atoms of the naphthalene ring and phenol units are marked with circle/dashed line and triangles/dotted line respectively. Blue, green and red signals are related to 1,5-alternate (1), 1,3,5-alternate (1) and 1,3,5,7-alternate conformation respectively; Figure S13: 2D-HSQC spectrum of **C_4_N_4_-Me** (CD_2_Cl_2_, 600 MHz, 193 K); Figure S14: Schematization of the possible conformers of **C_4_N_4_-Me**; Table S1: energy of the possible conformers of **C_4_N_4_-Me** obtained by molecular dynamics simulation; Figure S15: Structure of (a) 1,3,5,7- alternate (b) 1,3,5-alternate (1) and (c) 1,5-alternate (1) conformation obtained by molecular dynamics calculation; Table S2 - Crystal Data and Details of the Structure Determination for **C_4_N_4_-Me** and **C_4_N_4_** (CCDC deposit numbers = 2373754 and 2373755, respectively); Figure S16: ORTEP drawing of (a) **C_4_N_4_-Me** and (b) **C_4_N_4_**. Ellipsoids at 50% probability for the anisotropic thermal factors. Hydrogen atoms refined isotropically are shown in ball-and-stick representation; Table S3: Dihedral angles, θ, between the mean plane of the bridging methylene groups and the mean planes of the calixarene and naphthalene rings of **C_4_N_4_-Me** and **C_4_N_4_**; Figure S17: Top view of **C_4_N_4_-Me**, illustrating the hydrogen bonds between the donor hydroxy groups of the calixarene moieties and the acceptor alkoxy oxygens of the naphthalene moieties. The four hydrogen bonds are composed of two identical pairs, related by inversion through the centre of inversion located at the centre of the macrocycle, illustrated in green. Atoms are shown with CPK colours; Figure S18: Beer-Lambert plots for the determination of the extinction coefficients of **C_4_N_4_-Me**; Figure S19: Relative quantum yields for **C_4_N_4_-Me** (in hexane after excitation at 289 nm) were determined using anthracene (in ethanol) as standards; Table S4: Experimental values of interproton distances; Figure S20: Chemical drawing of 1,3,5-alternate (I) conformation of **C_4_N_4_-Me** obtained by molecular dynamics calculation; Table S5: Experimental values of interproton distances; Figure S21: Chemical drawing of 1, 5-alternate (I) conformation of **C_4_N_4_-Me** obtained by molecular dynamics calculation.
