# Supplementary material for: Deep-Cavity Calix[4]naphth[4]arene Macrocycles: Synthesis, Conformational Features, and Solid-State Structures
Source: Molecules. 2024 Aug 31;29(17):4142. doi: 10.3390/molecules29174142 (PMC11396966; doi:10.3390/molecules29174142)
Supplement: Supplementary file 1 [file molecules-29-04142-s001.zip › molecules-3165340-supplementary/Nuova cartella/2373754_CP82-data_platon_sq_file002.pdf]

No syntax errors found.
Please wait while processing ....

CIF dictionary
Interpreting this report

Datablock: platon_sq

Bond precision:	C-C = 0.0036 A		Wavelength=0.70000
Cell:	a=37.144(4)	b=9.674(1)	c=28.767(3)
	alpha=90	beta=113.448(9)	gamma=90
Temperature :	100 K		

	Calculated	Reported
Volume	9483.3(18)	9483.3(18)
Space group	C 2/c	C 2/c
Hall group	-C 2yc	-C 2yc
Moiety formula	C100 H112 O12 [+ solvent]	C100 H112 O12, 2.7(C H2 Cl2)
Sum formula	C100 H112 O12 [+ solvent]	C102.70 H117.40 Cl5.40 O12
Mr	1505.90	1735.19
Dx,g cm-3	1.055	1.215
Z	4	4
Mu (mm-1)	0.065	0.221
F000	3232.0	3686.0
F000'	3233.33	
h,k,lmax	52,13,40	52,13,38
Nref	14147	13143
Tmin,Tmax	0.987,0.996	
Tmin'	0.978	

Correction method= Not given

Data completeness= 0.929 Theta(max)= 29.740

R(reflections)= 0.0752(10617) wR2(reflections)= 0.2379(13143)

S = 1.029 Npar= 518

The following ALERTS were generated. Each ALERT has the format
test-name_ALERT_alert-type_alert-level.
Click on the hyperlinks for more details of the test.

Alert level B

PLAT097_ALERT_2_B Large Reported Max. (Positive) Residual Density 1.02 eA-3

Alert level C

PLAT029_ALERT_3_C _diffrn_measured_fraction_theta_full value Low . 0.969 Why?
PLAT052_ALERT_1_C Info on Absorption Correction Method Not Given Please Do !
PLAT213_ALERT_2_C Atom C122 has ADP max/min Ratio ..... 3.1 prolat
PLAT213_ALERT_2_C Atom C123 has ADP max/min Ratio ..... 3.7 prolat
PLAT220_ALERT_2_C NonSolvent Resd 1 C Ueq(max)/Ueq(min) Range 5.2 Ratio
PLAT222_ALERT_3_C NonSolvent Resd 1 H Uiso(max)/Uiso(min) Range 5.9 Ratio
PLAT230_ALERT_2_C Hirshfeld Test Diff for C145 --C147 . 5.5 s.u.
PLAT234_ALERT_4_C Large Hirshfeld Difference C120 --C123 . 0.16 Ang.
PLAT242_ALERT_2_C Low 'MainMol' Ueq as Compared to Neighbors of C120 Check
PLAT242_ALERT_2_C Low 'MainMol' Ueq as Compared to Neighbors of C145 Check
PLAT360_ALERT_2_C Short C(sp3)-C(sp3) Bond C120 - C122 . 1.40 Ang.
PLAT360_ALERT_2_C Short C(sp3)-C(sp3) Bond C120 - C123 . 1.43 Ang.
PLAT412_ALERT_2_C Short Intra XH3 .. XHn H12A ..H12E . 1.87 Ang.
x,y,z = 1_555 Check
PLAT906_ALERT_3_C Large K Value in the Analysis of Variance ...... 3.604 Check
PLAT911_ALERT_3_C Missing FCF Refl Between Thmin & STh/L= 0.600 265 Report

```
  0  2  0,   3  1  0,   7  1  0,   9  1  0,  13  5  0,  16  0  0,
-16  2  1,  -9  5  1,  -1  3  1,   4  2  1,   9  1  1, -15  1  2,
-10  0  2,  -7  3  2,  -2  0  2,   0  0  2,  22  0  2, -13  1  3,
  1  1  3,   4  2  3,  -4  0  4,  -3  3  4,  -2  0  4,  -1  3  4,
 -1  5  4,   2  0  4,   5  5  4, -25  3  5, -20  2  5,  -7  1  5,
 -2  0  6,   2  2  6,   5  1  6,   5  3  6,  -6  2  7,  -8  0  8,
 -4  0  8,  -4  2  8, -19  5 10, -16  4 10, -10  0 10,  -4  0 10,
```

```
                 4   0 10,  -4   4 11, -14   0 12,  -6   0 12,  -6   2 12,  -4   0 12,
                -8   0 14,  -6   0 14,  -4   0 14,  -4   2 15, -22   0 16,  -8   0 16,
                -6   0 16,  -4   0 16, -14   0 18, -10   0 18,  -8   0 18,  -6   0 18,
                -4   0 18,   0   0 18, -12   0 20, -10   0 20,  -8   0 20,  -6   0 20,
                -4   0 20, -16   0 22, -14   0 22, -12   0 22, -10   0 22,  -8   0 22,
                -6   0 22,  -4   0 22, -18   0 24, -16   0 24, -14   0 24, -13   1 24,
               -12   0 24, -11   1 24, -10   0 24,  -9   1 24,  -8   0 24,  -6   0 24,
                -4   0 24,  -2   0 24, -15   1 25, -13   1 25, -11   1 25,  -9   1 25,
                -7   1 25, -20   0 26, -18   0 26, -16   0 26, -15   1 26, -14   0 26,
PLAT918_ALERT_3_C Reflection(s) with I(obs) much Smaller I(calc) .          1 Check
PLAT977_ALERT_2_C Check Negative Difference Density on H12B       .     -0.42 eA-3
```

Alert level G

```
FORMU01_ALERT_2_G  There is a discrepancy between the atom counts in the
            _chemical_formula_sum and the formula from the _atom_site* data.
            Atom count from _chemical_formula_sum:C102.7 H117.4 Cl5.4 O12
            Atom count from the _atom_site data:  C100 H112 O12
ABSMU01_ALERT_1_G  Calculation of _exptl_absorpt_correction_mu
                not performed for this radiation type.
CELLZ01_ALERT_1_G Difference between formula and atom_site contents detected.
CELLZ01_ALERT_1_G ALERT: Large difference may be due to a
            symmetry error - see SYMMG tests
           From the CIF: _cell_formula_units_Z     4
           From the CIF: _chemical_formula_sum  C102.70 H117.40 Cl5.40 O12
           TEST: Compare cell contents of formula and atom_site data

           atom    Z*formula  cif sites diff
           C         410.80    400.00   10.80
           H         469.60    448.00   21.60
           Cl         21.60      0.00   21.60
           O          48.00     48.00    0.00
PLAT041_ALERT_1_G Calc. and Reported SumFormula    Strings  Differ     Please Check
              Calc: C100 H112 O12
              Rep.: C102.70 H117.40 Cl5.40 O12
PLAT042_ALERT_1_G Calc. and Reported MoietyFormula Strings  Differ     Please Check
              Calc: C100 H112 O12
              Rep.: C100 H112 O12, 2.7(C H2 Cl2)
PLAT051_ALERT_1_G Mu(calc) and Mu(CIF) Ratio Differs from 1.0 by .      70.57 %
PLAT083_ALERT_2_G SHELXL Second Parameter in WGHT  Unusually Large       6.70 Why ?
PLAT128_ALERT_4_G Alternate Setting for Input Space Group    C2/c        I2/a Note
PLAT605_ALERT_4_G Largest Solvent Accessible VOID in the Structure        178 A**3
PLAT869_ALERT_4_G ALERTS Related to the Use of SQUEEZE  Suppressed          ! Info
PLAT883_ALERT_1_G No Info/Value for _atom_sites_solution_primary .     Please Do !
PLAT899_ALERT_4_G SHELXL2018 is Deprecated and Succeeded by SHELXL     2019/3 Note
PLAT912_ALERT_4_G Missing # of FCF Reflections Above STh/L=  0.600        677 Note
PLAT913_ALERT_3_G Missing # of Very Strong Reflections in FCF ....          1 Note
                -2   0  4,
PLAT933_ALERT_2_G Number of HKL-OMIT Records in Embedded .res File         41 Note
                 2   0  4,   7   1  0,  -1   3  1,   2   2  6,  -7   3  2,  -6   2  7,
                16   0  0, -16   2  1, -10   0  2,   5   1  6,   0   2  0,   3   1  0,
                 4   2  1,   4   0 10, -18   0 24,   5   5  4,   9   1  1,   1   1  3,
               -15   1  2,  -9   5  1, -20   2  5,  -8   0  8, -25   3  5, -10   0 10,
               -16   4 10,  -3   3  4,  -6   2 12,  -7   1  5, -13   1  3, -14   0 12,
                 4   2  3,  -4   2  8,  13   5  0,   5   3  6,  -4   0  4, -22   0 16,
               -14   0 18,  -1   5  4,  -4   4 11,   9   1  0, -19   5 10,
PLAT952_ALERT_5_G Calculated (ThMax) and CIF-Reported Lmax Differ.          2 Units
PLAT958_ALERT_1_G Calculated (ThMax) and Actual (FCF) Lmax Differ.          2 Units
PLAT969_ALERT_5_G The 'Henn et al.' R-Factor-gap value ..........      11.276 Note
              Predicted wR2: Based on SigI**2  2.11 or SHELX Weight 23.18
PLAT978_ALERT_2_G Number C-C Bonds with Positive Residual Density.          4 Info
PLAT992_ALERT_5_G Repd & Actual _reflns_number_gt Values Differ by          2 Check
```

```
  0 ALERT level A = Most likely a serious problem - resolve or explain
  1 ALERT level B = A potentially serious problem, consider carefully
 17 ALERT level C = Check. Ensure it is not caused by an omission or oversight
 21 ALERT level G = General information/check it is not something unexpected

  9 ALERT type 1 CIF construction/syntax error, inconsistent or missing data
 15 ALERT type 2 Indicator that the structure model may be wrong or deficient
```

```
   6 ALERT type 3 Indicator that the structure quality may be low
   6 ALERT type 4 Improvement, methodology, query or suggestion
   3 ALERT type 5 Informative message, check
```

It is advisable to attempt to resolve as many as possible of the alerts in all categories. Often the minor alerts point to easily fixed oversights, errors and omissions in your CIF or refinement strategy, so attention to these fine details can be worthwhile. In order to resolve some of the more serious problems it may be necessary to carry out additional measurements or structure refinements. However, the purpose of your study may justify the reported deviations and the more serious of these should normally be commented upon in the discussion or experimental section of a paper or in the "special_details" fields of the CIF. checkCIF was carefully designed to identify outliers and unusual parameters, but every test has its limitations and alerts that are not important in a particular case may appear. Conversely, the absence of alerts does not guarantee there are no aspects of the results needing attention. It is up to the individual to critically assess their own results and, if necessary, seek expert advice.

Publication of your CIF in IUCr journals

A basic structural check has been run on your CIF. These basic checks will be run on all CIFs submitted for publication in IUCr journals (*Acta Crystallographica*, *Journal of Applied Crystallography*, *Journal of Synchrotron Radiation*); however, if you intend to submit to *Acta Crystallographica Section C* or *E* or *IUCrData*, you should make sure that full publication checks are run on the final version of your CIF prior to submission.

Publication of your CIF in other journals

Please refer to the *Notes for Authors* of the relevant journal for any special instructions relating to CIF submission.

PLATON version of 15/07/2024; check.def file version of 15/07/2024

Datablock platon_sq - ellipsoid plot

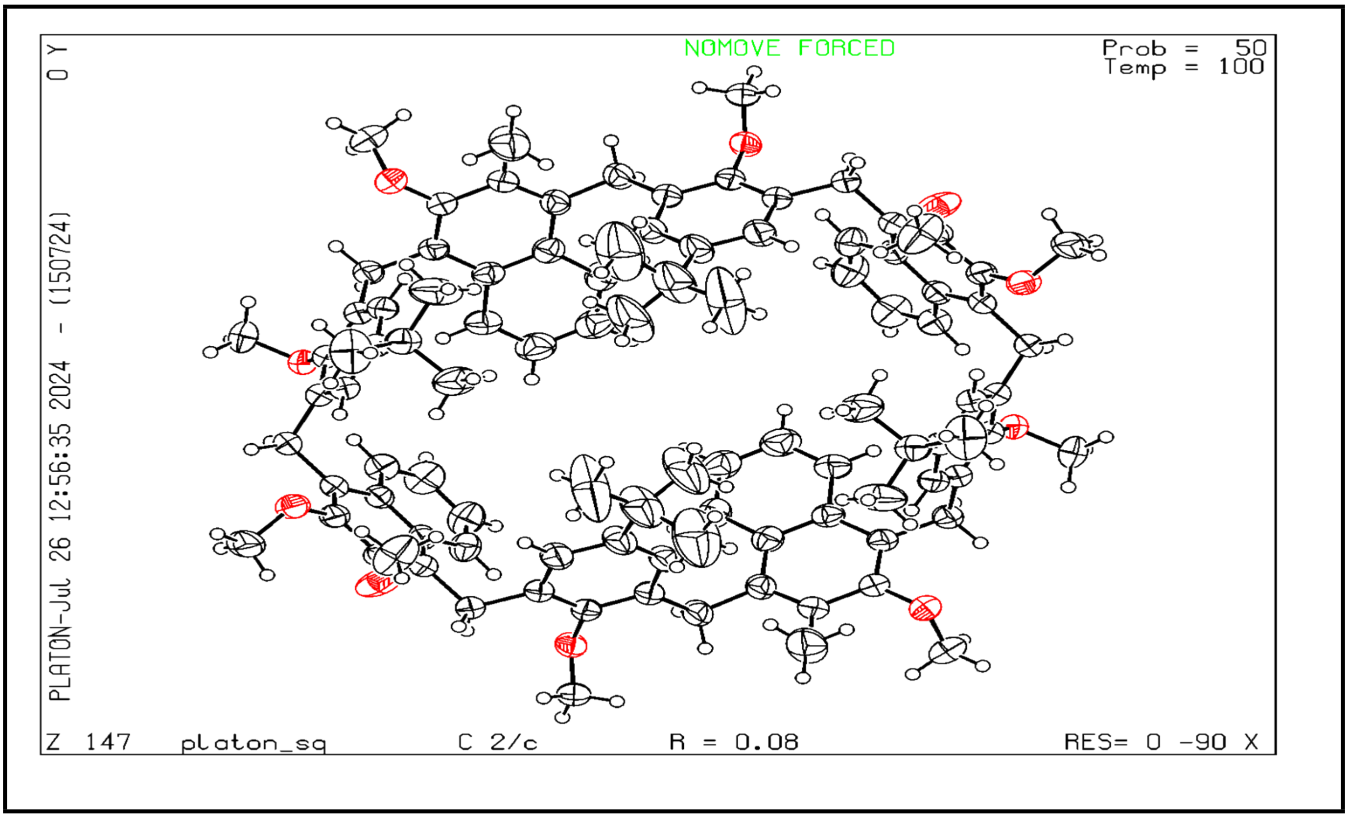

Download CIF editor (publCIF) from the IUCr
Download CIF editor (enCIFer) from the CCDC
Test a new CIF entry
