# Supplementary material for: Deep-Cavity Calix[4]naphth[4]arene Macrocycles: Synthesis, Conformational Features, and Solid-State Structures
Source: Molecules. 2024 Aug 31;29(17):4142. doi: 10.3390/molecules29174142 (PMC11396966; doi:10.3390/molecules29174142)
Supplement: Supplementary file 1 [file molecules-29-04142-s001.zip › molecules-3165340-supplementary/Nuova cartella/2373755_CP62-data_platon_sq_file004.pdf]

No syntax errors found. [CIF dictionary](#)
Please wait while processing .... [Interpreting this report](#)

Datablock: platon_sq

Bond precision: C-C = 0.0041 A Wavelength=0.70000
Cell: a=9.593(8) b=13.908(3) c=18.235(5)
alpha=113.342(3) beta=94.802(11) gamma=97.707(5)
Temperature : 100 K

	Calculated	Reported
Volume	2188(2)	2188(2)
Space group	P -1	P -1
Hall group	-P 1	-P 1
Moiety formula	C96 H104 O12 [+ solvent]	C96 H104 O12, 2.2(C H2 Cl2)
Sum formula	C96 H104 O12 [+ solvent]	C98.20 H108.40 Cl4.40 O12
Mr	1449.79	1636.62
Dx,g cm-3	1.100	1.242
Z	1	1
Mu (mm-1)	0.068	0.206
F000	776.0	868.0
F000'	776.32	
h,k,lmax	12,18,24	12,18,24
Nref	10832	9957
Tmin,Tmax	0.988,0.996	
Tmin'	0.980	

Correction method= Not given
Data completeness= 0.919 Theta(max)= 27.789
R(reflections)= 0.0642(5818) wR2(reflections)= 0.2038(9957)
S = 1.036 Npar= 499

The following ALERTS were generated. Each ALERT has the format
test-name_ALERT_alert-type_alert-level.
Click on the hyperlinks for more details of the test.

Alert level A

PLAT029_ALERT_3_A _diffrn_measured_fraction_theta_full value Low . 0.933 Why?

Author Response: The unit cell under investigation is triclinic (space group = P-1). The low value is therefore due to a missing cusp of data, as is often the case for this space group. The refined structure has a data-to-parameter ratio of 11.6 (20.0) (5818 (9957) independent reflections (Fo > 4sig(Fo), 499 parameters) and the quality of the model can be considered as sufficient for the purposes of the present study.

Alert level C

PLAT052_ALERT_1_C Info on Absorption Correction Method Not Given Please Do !
PLAT155_ALERT_4_C The Triclinic Unitcell is NOT Reduced .... Please Do !
PLAT242_ALERT_2_C Low 'MainMol' Ueq as Compared to Neighbors of C144 Check
PLAT340_ALERT_3_C Low Bond Precision on C-C Bonds .... 0.00406 Ang.
PLAT905_ALERT_3_C Negative K value in the Analysis of Variance ... -3.549 Report
PLAT911_ALERT_3_C Missing FCF Refl Between Thmin & STh/L= 0.600 530 Report

-3 8 0, 1-13 1, 0 -2 1, 6 -2 1, 0 -1 1, 3 0 1,
-7 2 1, -6-11 2, -3 -6 2, 0 -3 2, 6 -2 2, -6 4 2,
-2-15 3, -1-15 3, 0-15 3, -3-14 3, -2-14 3, -1-14 3,
-2-13 3, -1-13 3, 9-10 3, -1 -7 3, -1 -6 3, -1 -5 3,
0 -5 3, -1 -4 3, 0 -4 3, 6 -4 3, -3 0 3, 0-16 4,
1-16 4, -2-15 4, -1-15 4, 0-15 4, 1-15 4, -4-14 4,
-3-14 4, -2-14 4, -1-14 4, 0-14 4, 1-14 4, -3-13 4,
-2-13 4, -1-13 4, 0-13 4, -3-12 4, -2-12 4, -1-12 4,
0-12 4, -2-11 4, -1-11 4, 0-11 4, -2-10 4, -1-10 4,
-2 -9 4, -1 -9 4, 0 -9 4, -1 -8 4, 0 -8 4, -1 -7 4,

0 -7 4, -6 -6 4, -1 -6 4, 0 -6 4, -1 -5 4, 0 -5 4,
1 -2 4, 0-16 5, 1-16 5, 2-16 5, -3-15 5, -2-15 5,
-1-15 5, 0-15 5, 1-15 5, -4-14 5, -3-14 5, -2-14 5,
-1-14 5, 0-14 5, 1-14 5, -4-13 5, -3-13 5, -2-13 5,
-1-13 5, 0-13 5, 1-13 5, -4-12 5, -3-12 5, -2-12 5,
-1-12 5, 0-12 5, 1-12 5, -3-11 5, -2-11 5, -1-11 5,

Alert level G

FORMU01_ALERT_2_G There is a discrepancy between the atom counts in the _chemical_formula_sum and the formula from the _atom_site* data.
Atom count from _chemical_formula_sum:C98.2 H108.4 Cl4.4 O12
Atom count from the _atom_site data: C96 H104 O12

ABSMU01_ALERT_1_G Calculation of _exptl_absorpt_correction_mu not performed for this radiation type.

CELLZ01_ALERT_1_G Difference between formula and atom_site contents detected.

CELLZ01_ALERT_1_G ALERT: Large difference may be due to a symmetry error - see SYMMG tests
From the CIF: _cell_formula_units_Z 1
From the CIF: _chemical_formula_sum C98.20 H108.40 Cl4.40 O12
TEST: Compare cell contents of formula and atom_site data

atom	Z*formula	cif sites	diff
C	98.20	96.00	2.20
H	108.40	104.00	4.40
Cl	4.40	0.00	4.40
O	12.00	12.00	0.00

PLAT007_ALERT_5_G Number of Unrefined Donor-H Atoms .... 2 Report
H10A H103

PLAT041_ALERT_1_G Calc. and Reported SumFormula Strings Differ Please Check
Calc: C96 H104 O12
Rep.: C98.20 H108.40 Cl4.40 O12

PLAT042_ALERT_1_G Calc. and Reported MoietyFormula Strings Differ Please Check
Calc: C96 H104 O12
Rep.: C96 H104 O12, 2.2(C H2 Cl2)

PLAT051_ALERT_1_G Mu(calc) and Mu(CIF) Ratio Differs from 1.0 by . 66.88 %

PLAT605_ALERT_4_G Largest Solvent Accessible VOID in the Structure 175 A**3

PLAT869_ALERT_4_G ALERTS Related to the Use of SQUEEZE Suppressed ! Info

PLAT883_ALERT_1_G No Info/Value for _atom_sites_solution_primary . Please Do !

PLAT899_ALERT_4_G SHELXL2018 is Deprecated and Succeeded by SHELXL 2019/3 Note

PLAT912_ALERT_4_G Missing # of FCF Reflections Above STh/L= 0.600 345 Note

PLAT933_ALERT_2_G Number of HKL-OMIT Records in Embedded .res File 3 Note
1 -2 4, 2 -3 7, -1 -5 6,

PLAT941_ALERT_3_G Average HKL Measurement Multiplicity .... 3.4 Low

PLAT969_ALERT_5_G The 'Henn et al.' R-Factor-gap value .... 4.684 Note
Predicted wR2: Based on SigI**2 4.35 or SHELX Weight 19.74

PLAT978_ALERT_2_G Number C-C Bonds with Positive Residual Density. 0 Info

---

1 **ALERT level A** = Most likely a serious problem - resolve or explain
0 **ALERT level B** = A potentially serious problem, consider carefully
6 **ALERT level C** = Check. Ensure it is not caused by an omission or oversight
17 **ALERT level G** = General information/check it is not something unexpected

8 ALERT type 1 CIF construction/syntax error, inconsistent or missing data
4 ALERT type 2 Indicator that the structure model may be wrong or deficient
5 ALERT type 3 Indicator that the structure quality may be low
5 ALERT type 4 Improvement, methodology, query or suggestion
2 ALERT type 5 Informative message, check

It is advisable to attempt to resolve as many as possible of the alerts in all categories. Often the minor alerts point to easily fixed oversights, errors and omissions in your CIF or refinement strategy, so attention to these fine details can be worthwhile. In order to resolve some of the more serious problems it may be necessary to carry out additional measurements or structure refinements. However, the purpose of your study may justify the reported deviations and the more serious of these should

normally be commented upon in the discussion or experimental section of a paper or in the "special_details" fields of the CIF. checkCIF was carefully designed to identify outliers and unusual parameters, but every test has its limitations and alerts that are not important in a particular case may appear. Conversely, the absence of alerts does not guarantee there are no aspects of the results needing attention. It is up to the individual to critically assess their own results and, if necessary, seek expert advice.

Publication of your CIF in IUCr journals

A basic structural check has been run on your CIF. These basic checks will be run on all CIFs submitted for publication in IUCr journals (*Acta Crystallographica*, *Journal of Applied Crystallography*, *Journal of Synchrotron Radiation*); however, if you intend to submit to *Acta Crystallographica Section C* or *E* or *IUCrData*, you should make sure that full publication checks are run on the final version of your CIF prior to submission.

Publication of your CIF in other journals

Please refer to the *Notes for Authors* of the relevant journal for any special instructions relating to CIF submission.

PLATON version of 15/07/2024; check.def file version of 15/07/2024

Datablock platon_sq - ellipsoid plot

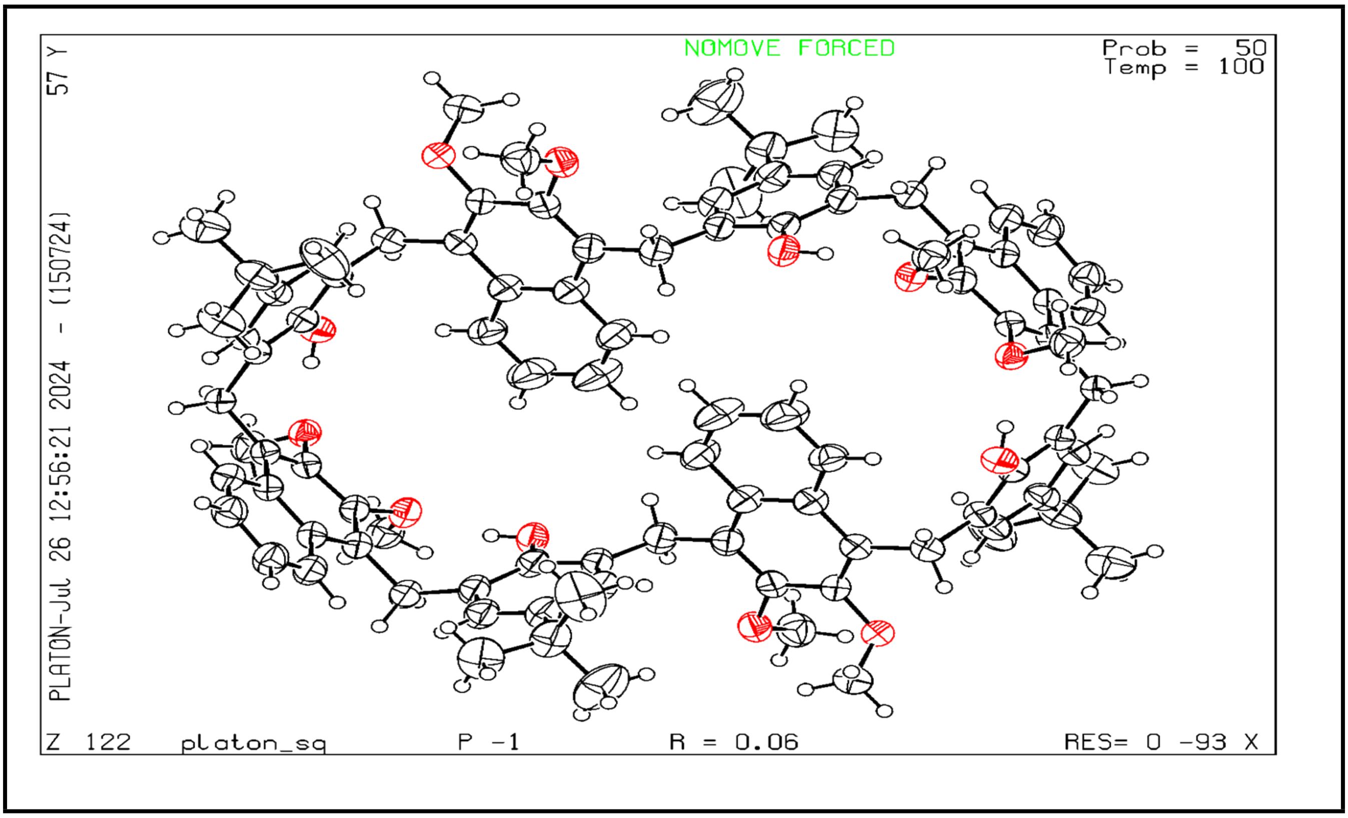

Download CIF editor (publCIF) from the IUCr
Download CIF editor (enCIFer) from the CCDC
Test a new CIF entry
