# Supplementary material for: Deep-Cavity Calix[4]naphth[4]arene Macrocycles: Synthesis, Conformational Features, and Solid-State Structures
Source: Molecules. 2024 Aug 31;29(17):4142. doi: 10.3390/molecules29174142 (PMC11396966; doi:10.3390/molecules29174142)
Supplement: Supplementary file 1 [file molecules-29-04142-s001.zip › molecules-3165340-supplementary/Nuova cartella/Supplementary Information.pdf]

Supplementary Information

Calix[4]Naphth[4]arene as a Novel Deep-Cavity Macrocycles: Synthesis, Conformational Features, and Solid-State Structures

Paolo Della Sala,[*,1] Veronica Iuliano,[1] Margherita De Rosa,[1] Carmen Talotta,[1] Rocco Del Regno,[1] Placido Neri,[1] Silvano Geremia,[2] Neal Hickey[2] and Carmine Gaeta[1]

[1] Laboratory of Supramolecular Chemistry, Dipartimento di Chimica e Biologia "A. Zambelli", Università degli Studi di Salerno, Via Giovanni Paolo II, 132, 84084, Fisciano, Salerno (Italy).

[2] Centro di Eccellenza in Biocristallografia, Dipartimento di Scienze Chimiche e Farmaceutiche, Università di Trieste, Via L. Giorgieri 1, 34127 Trieste (Italy).

Table of contents	Pages
Copies of NMR and Mass Spectra of **C_4N_4**	**S2 - S6**
Copies of NMR and Mass Spectra of **C_4N_4-Me**	**S7 - S11**
VT NMR studies of **C_4N_4-Me**	**S12 - S14**
Computational studies on **C_4N_4-Me** conformers	**S15 - S16**
Atomic coordinates of 1,5-Alternate (I) of **C_4N_4-Me**	**S17 - S20**
Atomic coordinates of 1,3,5-Alternate (I) of **C_4N_4-Me**	**S21 - S24**
Atomic coordinates of 1,3,5,7-Alternate of **C_4N_4-Me**	**S25 - S28**
Crystallographic structure determination of **C_4N_4-Me** and **C_4N_4**	**S29 - S31**
UV-Vis and Fluorescence Characterization of **C_4N_4-Me**	**S32**
Quantitative Distance Determination by NOESY experiment	**S33**

Copies of NMR and Mass Spectra of C_4N_4

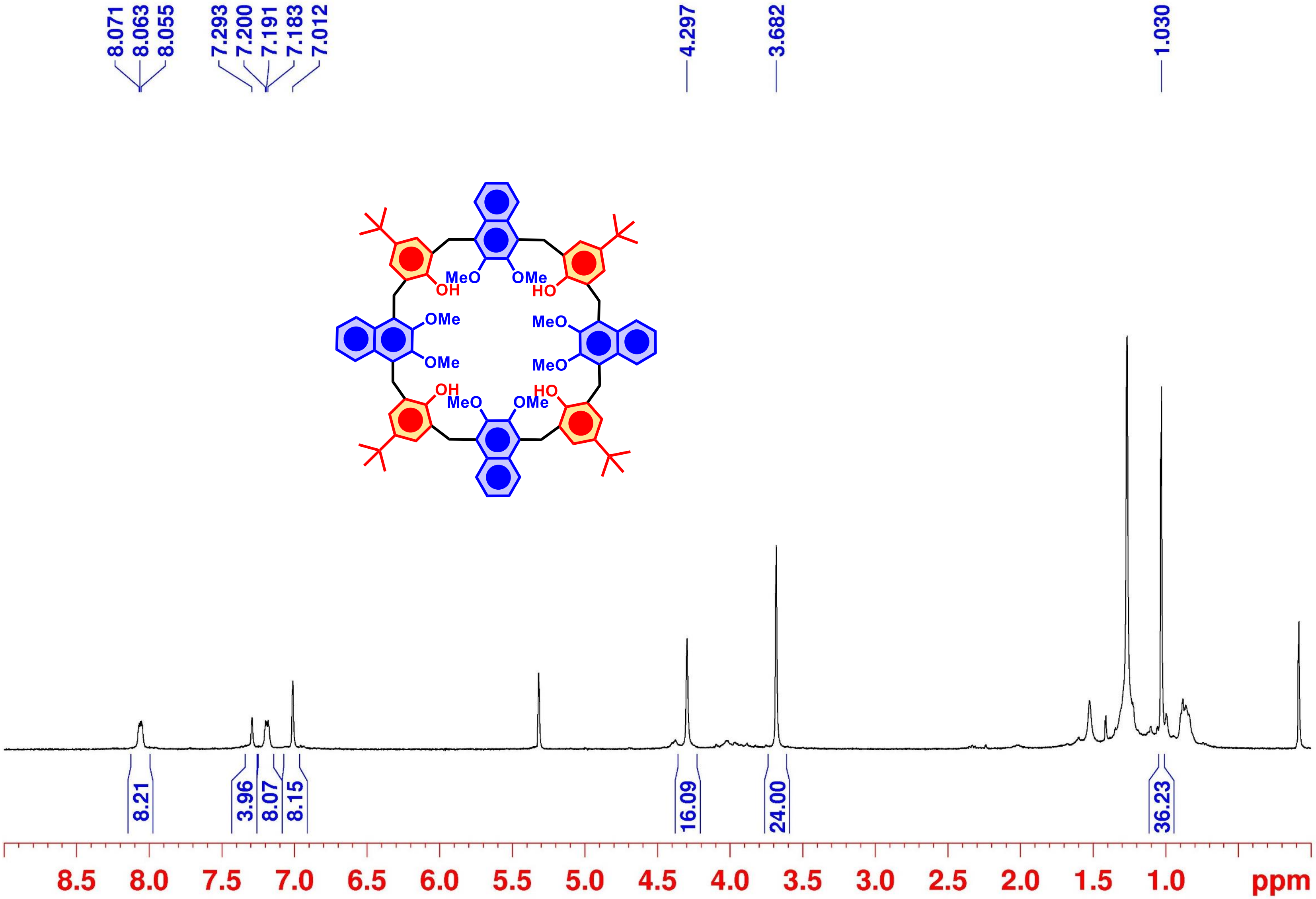

Figure S1: ^{1}H NMR spectrum of **C_4N_4** (CD_2Cl_2, 400 MHz, 298 K).

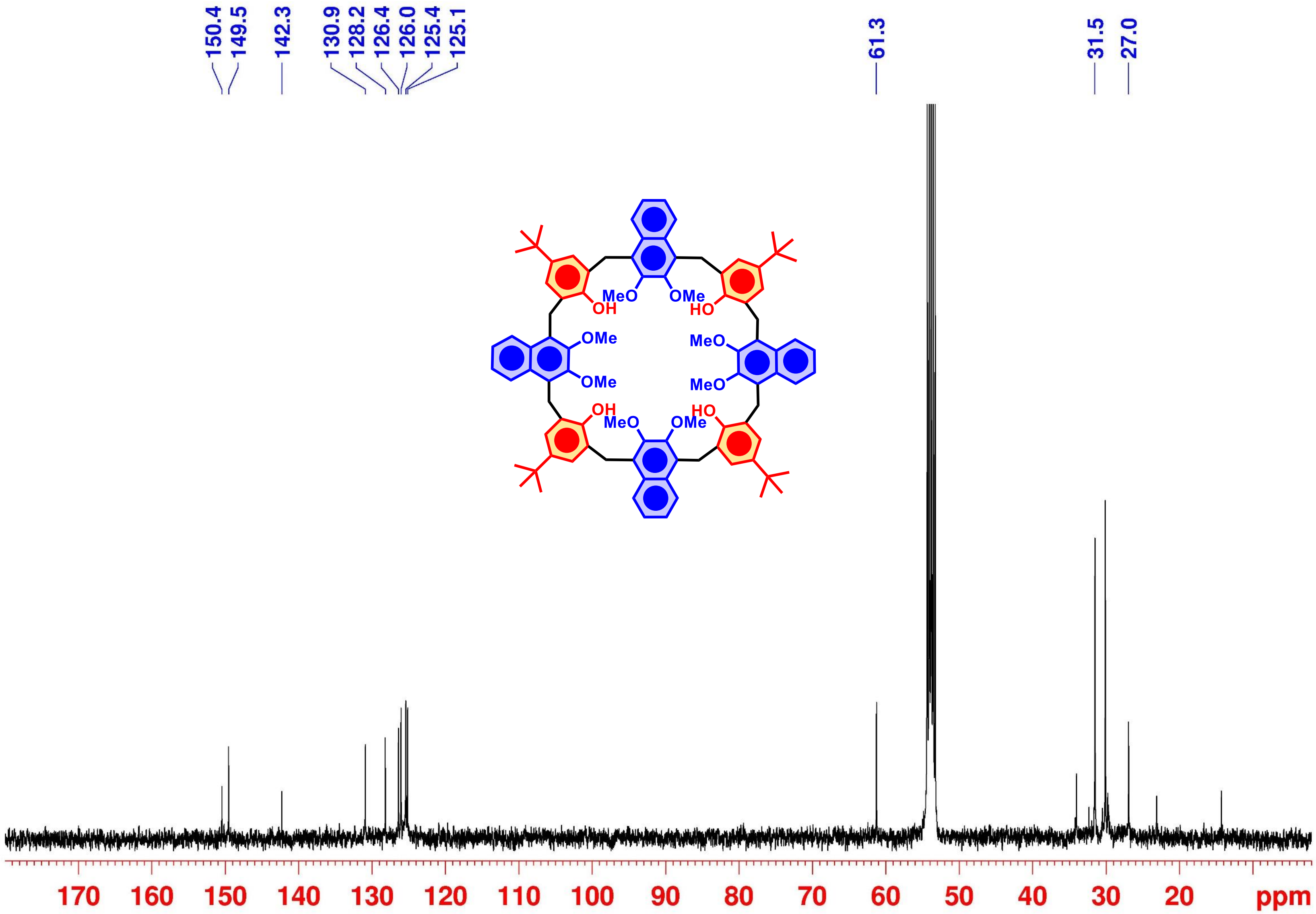

Figure S2: ^{13}C NMR {^{1}H} spectrum of **C_4N_4** (CD_2Cl_2, 100 MHz, 298 K).

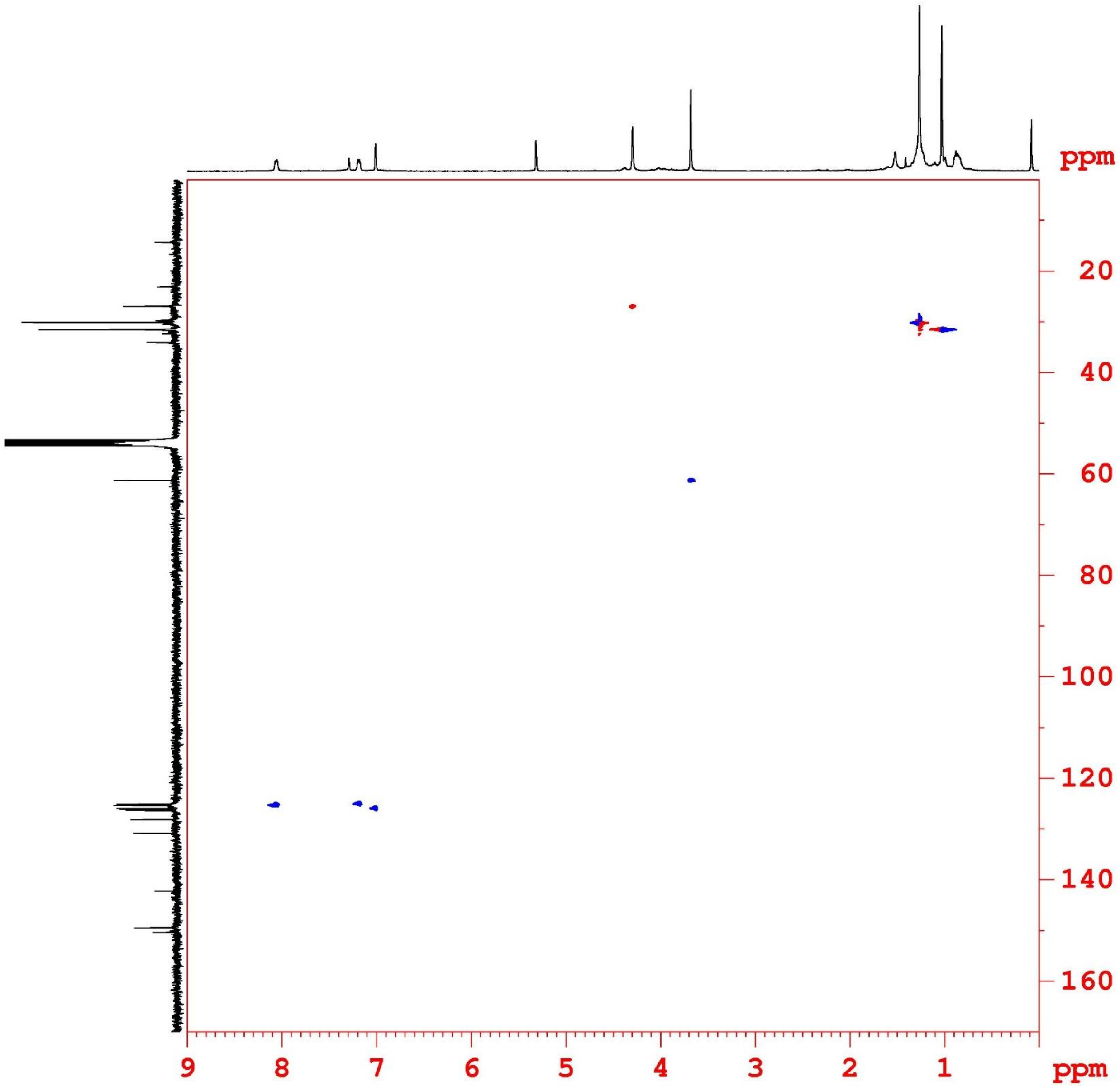

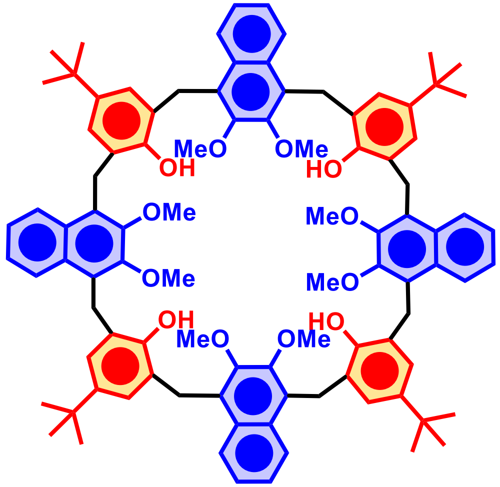

Figure S3: 2D-HSQC spectrum of **C_4N_4** (CD_2Cl_2, 400 MHz, 298 K).

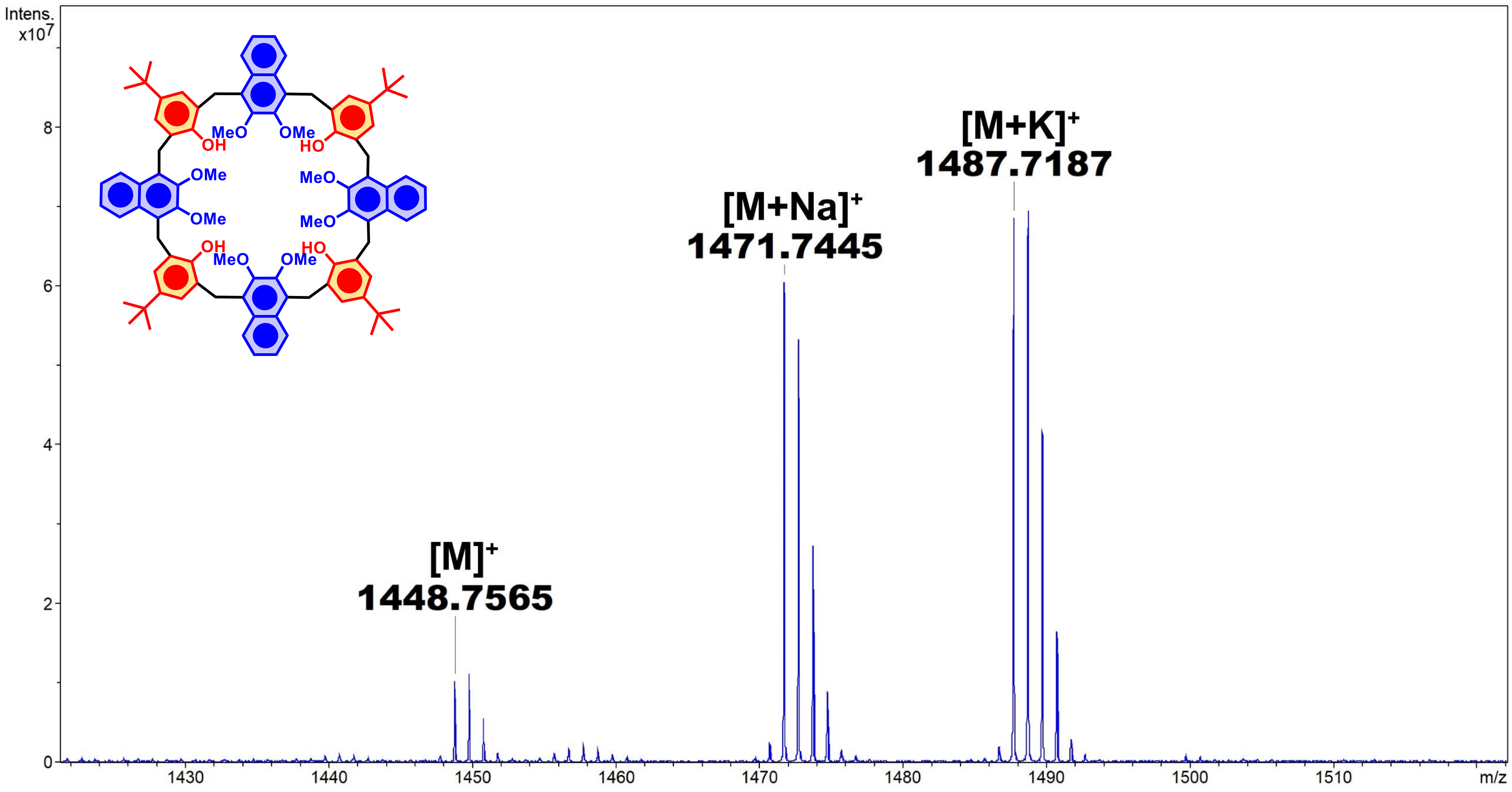

Figure S4: Significant portion of the HR MALDI FT-ICR mass spectrum of **C_4N_4-Me.**

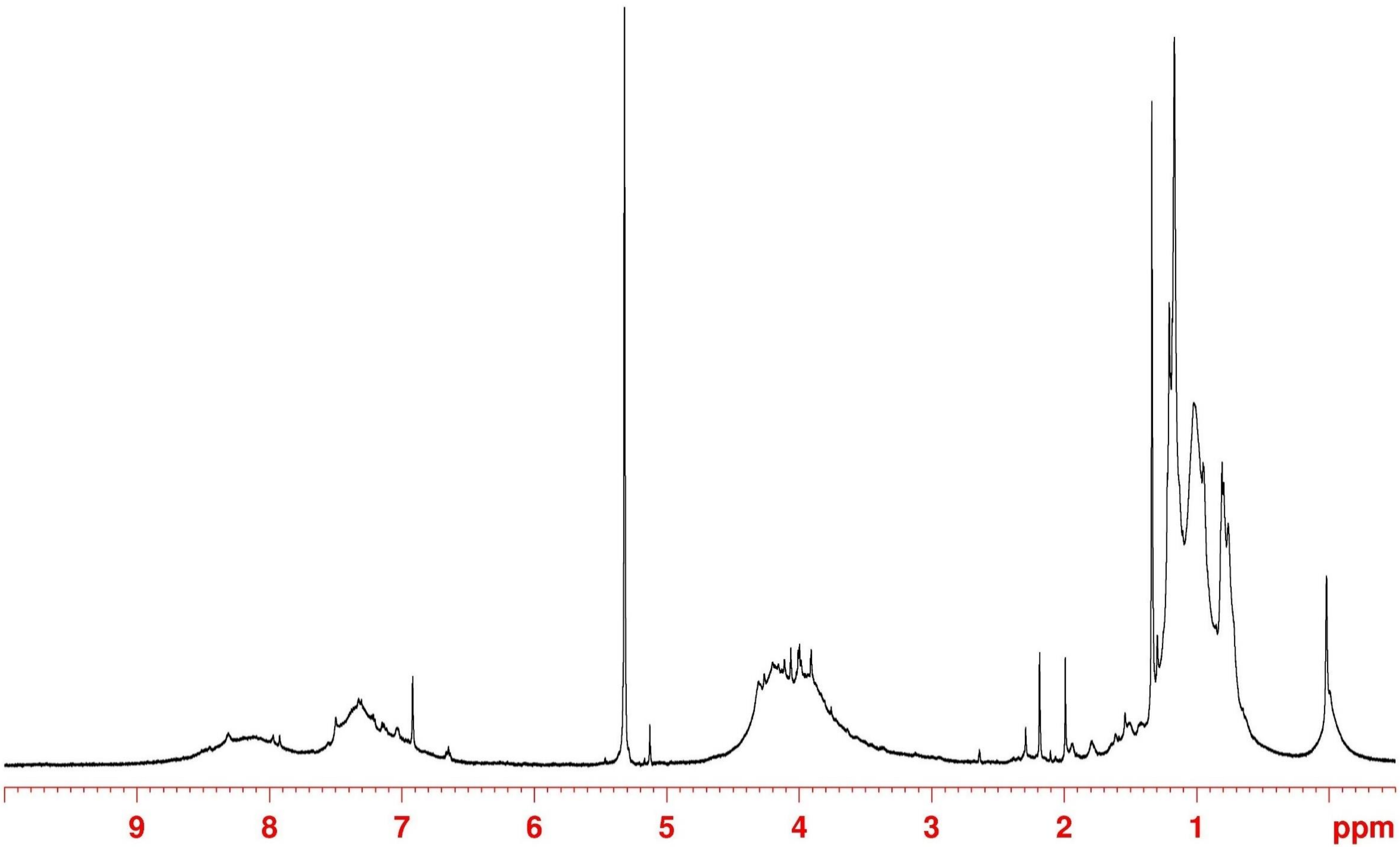

Figure S5: ^{1}H NMR spectrum of **C_4N_4** (CD_2Cl_2, 600 MHz, 183 K).

Copies of NMR and Mass Spectra of C_4N_4-Me

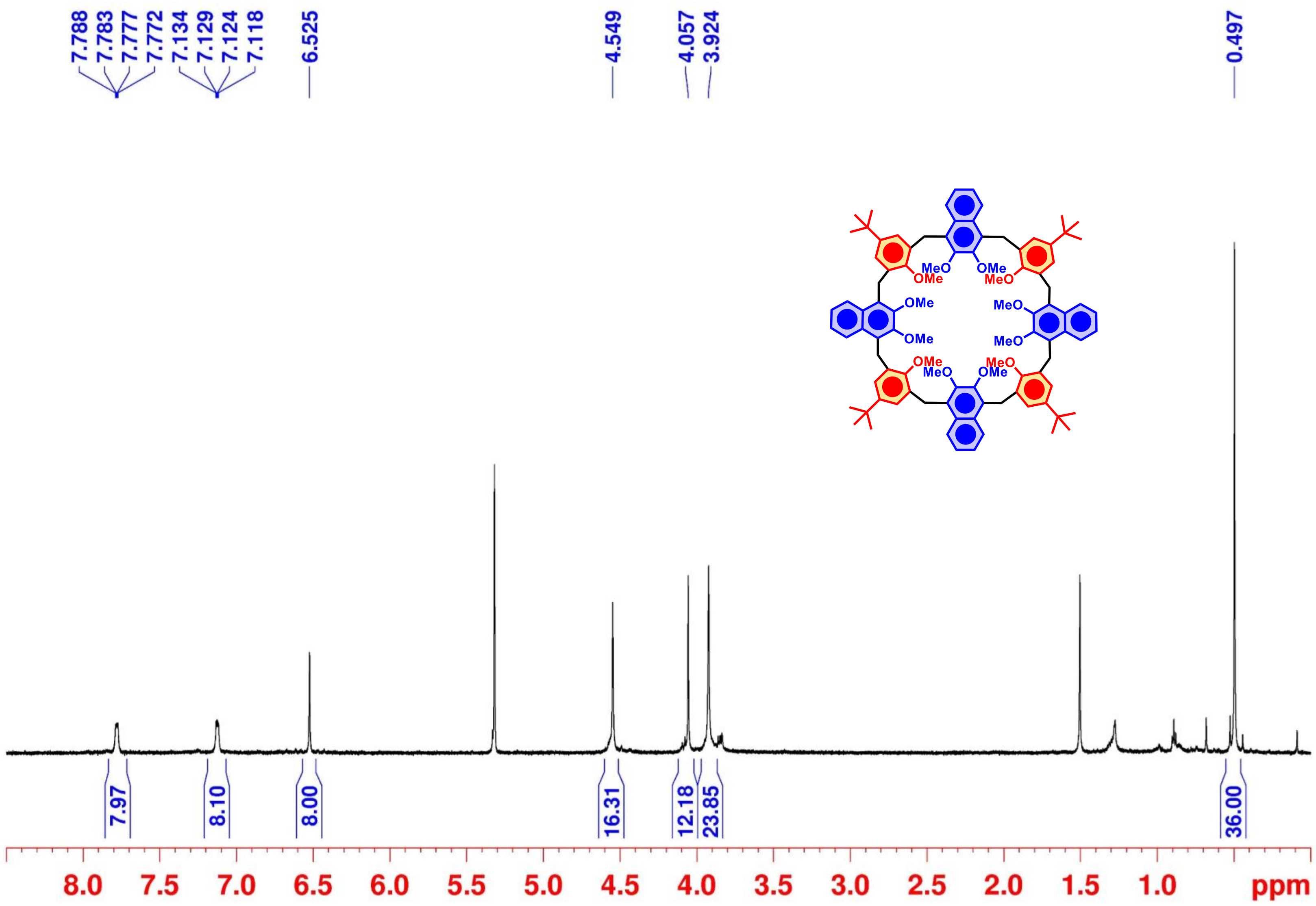

Figure S6: ^{1}H NMR spectrum of **C_4N_4-Me** (CD_2Cl_2, 600 MHz, 298 K).

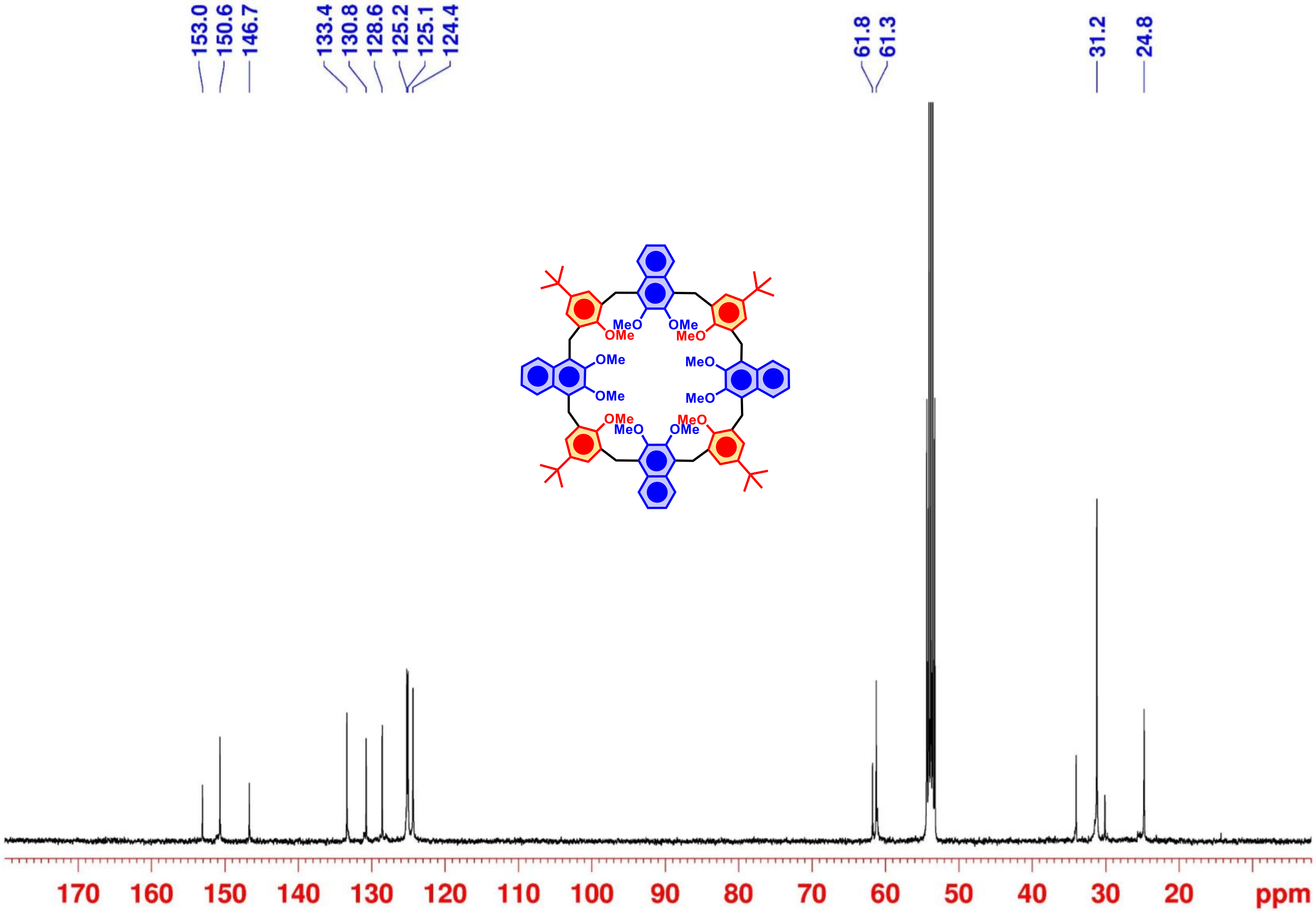

Figure S7: ^{13}C NMR {^{1}H} spectrum of **C_4N_4-Me** (CD_2Cl_2, 100 MHz, 298 K).

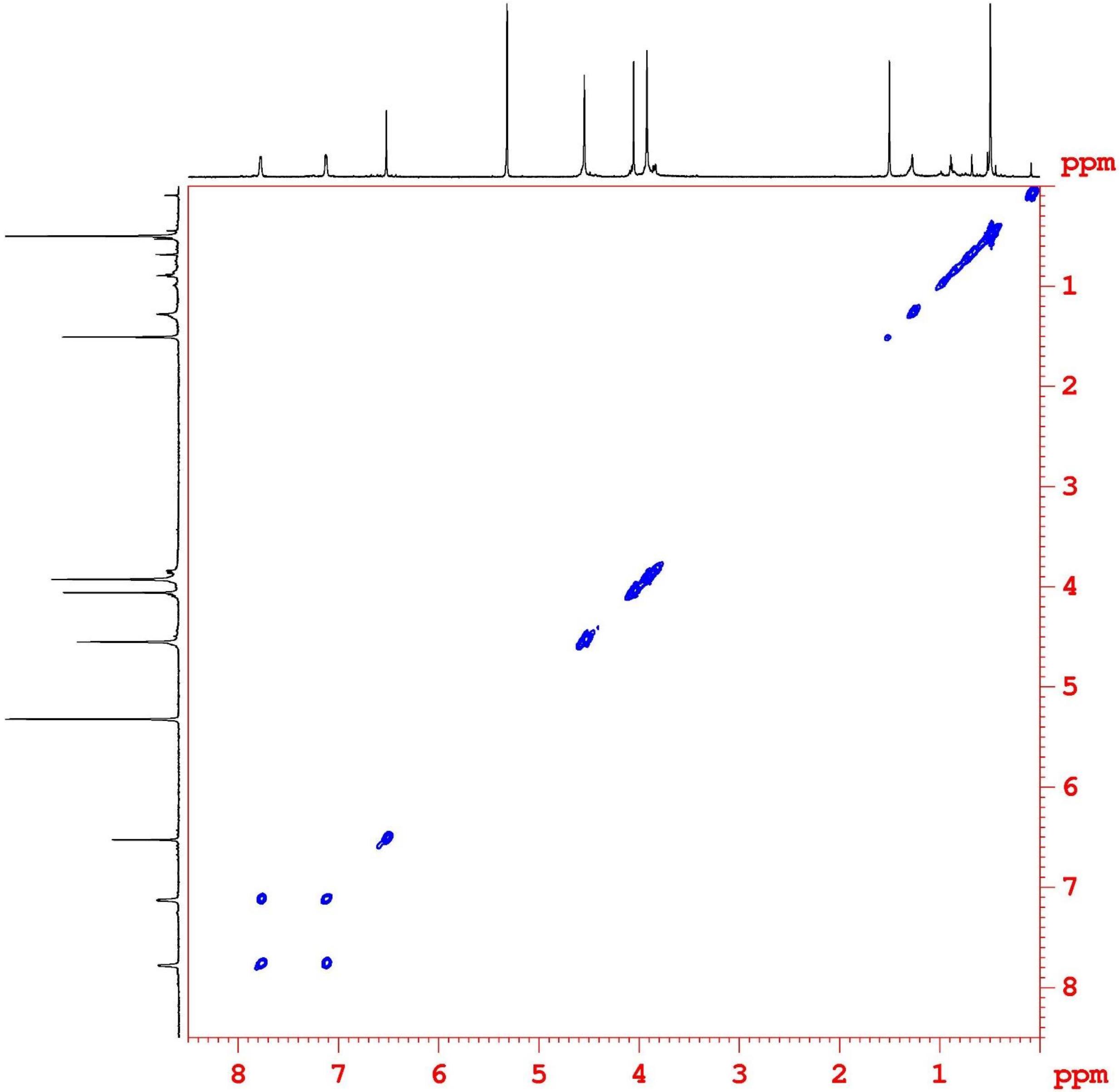

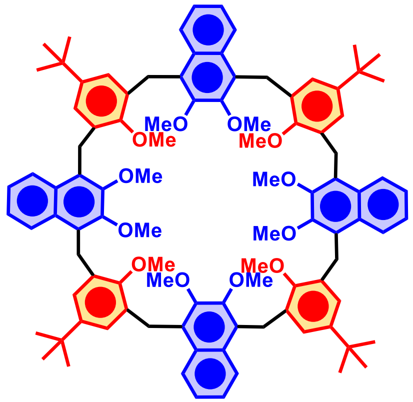

Figure S8: 2D-DQF COSY spectrum of **C_4N_4-Me** (CD_2Cl_2, 400 MHz, 298 K).

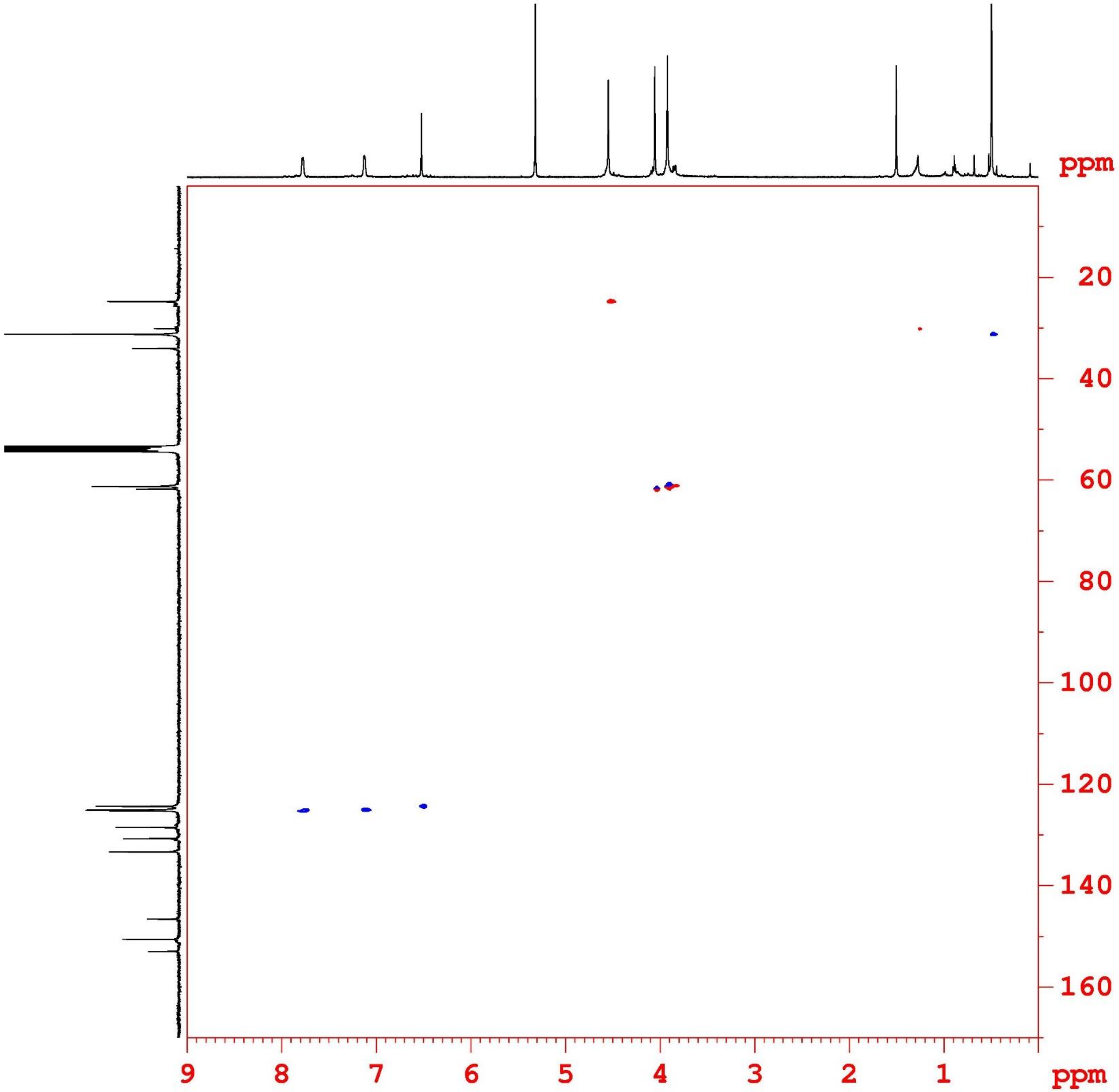

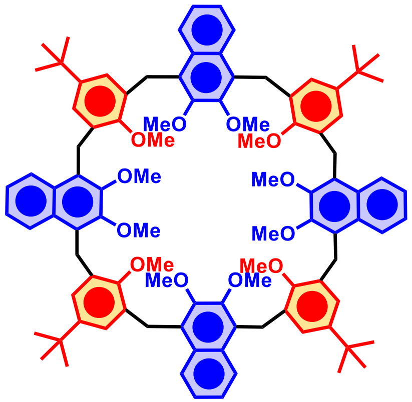

Figure S9: 2D-HSQC spectrum of **C_4N_4-Me** (CD_2Cl_2, 400 MHz, 298 K).

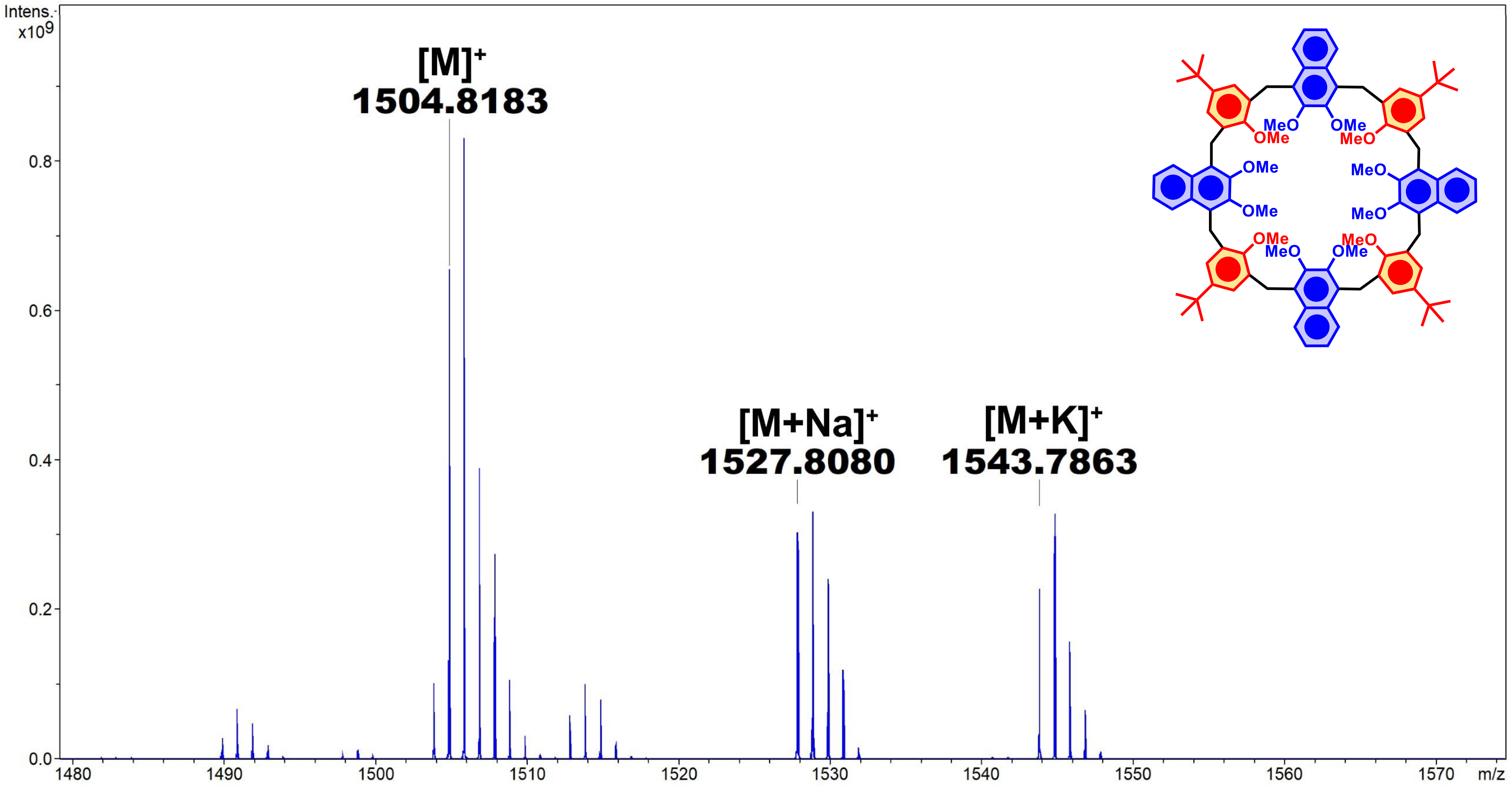

Figure S10: Significant portion of the HR MALDI FT-ICR mass spectrum of **C_4N_4-Me.**

VT NMR studies of C_4N_4-Me

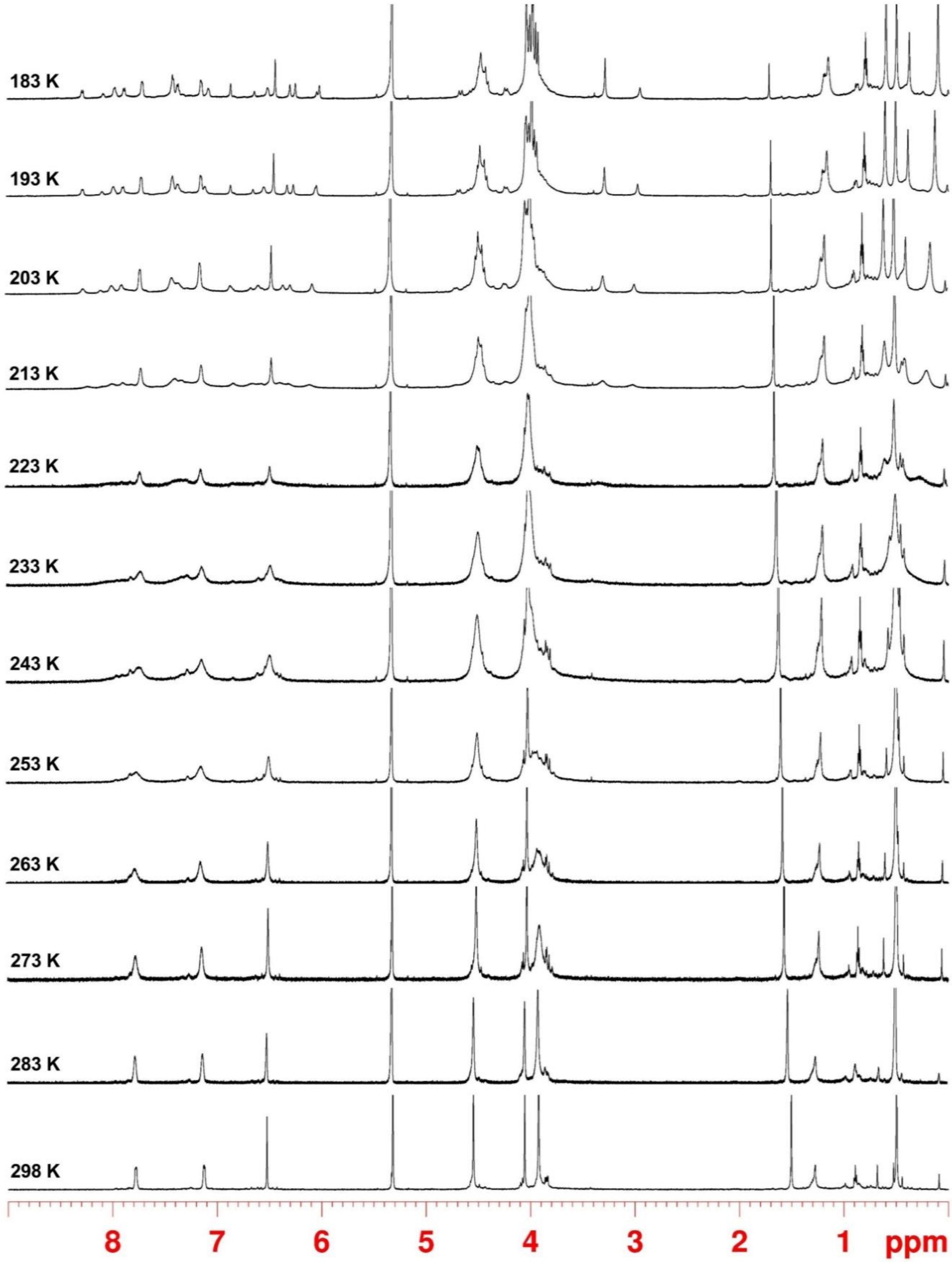

Figure S11: ^{1}H NMR spectra of **C_4N_4-Me** (CD_2Cl_2, 600 MHz) at (from bottom to top): 298, 283, 273, 263, 253, 243, 233, 223, 213, 203, 193 and 183 K.

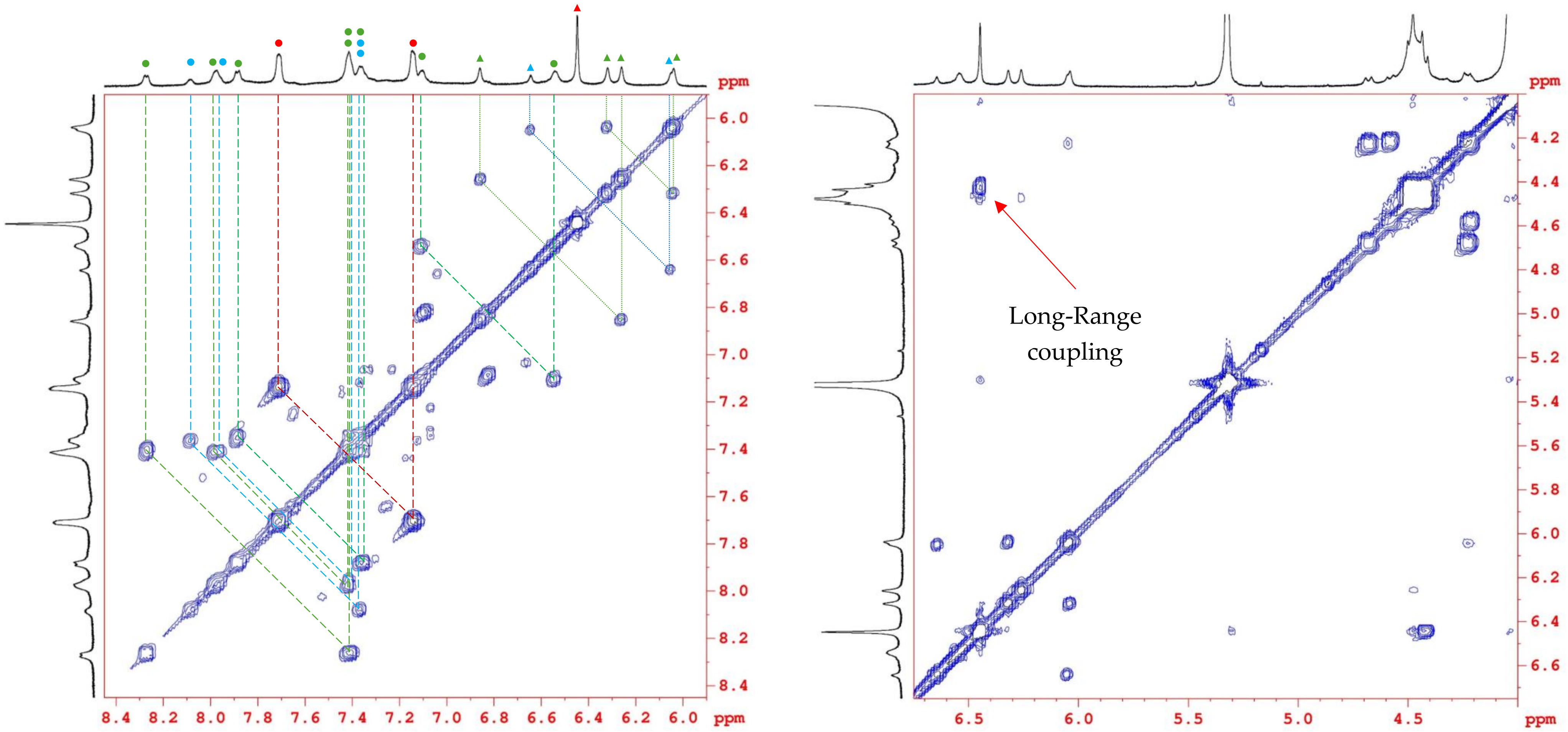

Figure S12: Portions of 2D-DQF COSY spectrum of **C_4N_4-Me** (CD_2Cl_2, 600 MHz, 193 K). The signals of the aromatic hydrogen atoms of the naphthalene ring and phenol units are marked with circle/dashed line and triangles/dotted line respectively. Blue, green and red signals are related to 1,5-alternate (1), 1,3,5-alternate (1) and 1,3,5,7-alternate conformation respectively.

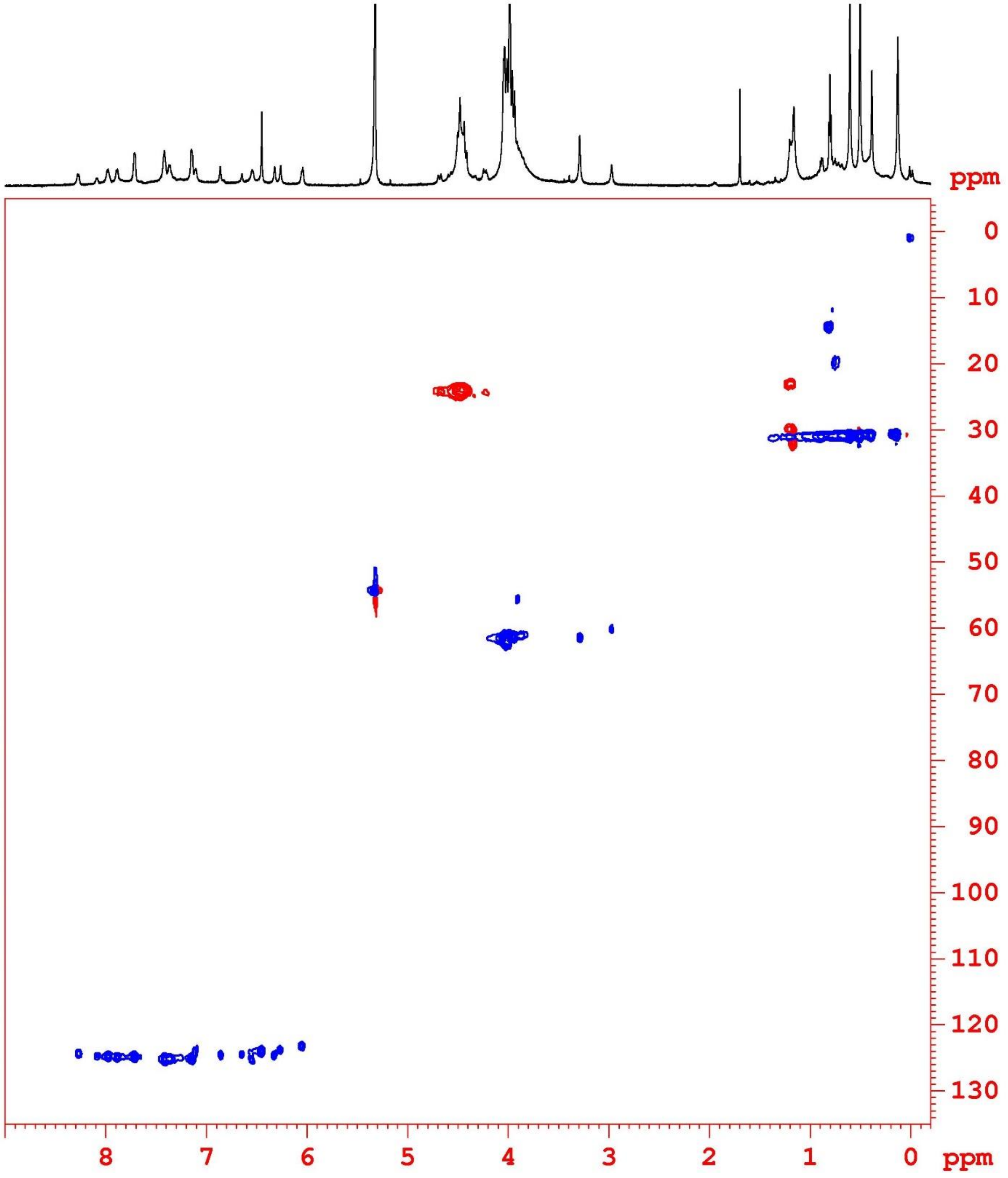

Figure S13: 2D-HSQC spectrum of **C_4N_4-Me** (CD_2Cl_2, 600 MHz, 193 K).

Computational studies on C_4N_4-Me conformers

Table S1

Conformer	Energy	ΔE
1,3,5-Alt (1)	93.61	0.00
1,3,5,7-Alt	93.73	0.12
1,5-Alt (1)	94.61	1.00
1,2,4,5-Alt (1)	95.10	1.49
1,2,4-Alt (2)	96.37	2.76
1,2,5,6-Alt	96.90	3.29
1,2,4,7-Alt	97.16	3.55
1,2,3-Alt (2)	97.36	3.75
1,4-Alt	97.45	3.84
1,2,4,5-Alt (2)	97.60	3.99
1,3-Alt (2)	98.40	4.79
1,3,6-Alt (2)	99.01	5.40
1,2-Alt	99.22	5.61
1,2,3,5-Alt	99.56	5.95
1,2,4-Alt (1)	99.73	6.12
1,2,3-Alt (1)	100.22	6.61
PaCo (2)	100.28	6.67
1,3,5-Alt (2)	100.78	7.17
1,2,3,4-Alt	100.95	7.34
1,3-Alt (1)	102.46	8.85
PaCo (1)	103.34	9.73
1,2,5-Alt (2)	103.38	9.77
1,5-Alt (2)	105.16	11.55
1,3,6-Alt (1)	105.90	12.29
1,2,5-Alt (1)	108.01	14.40
Cone	109.59	15.98

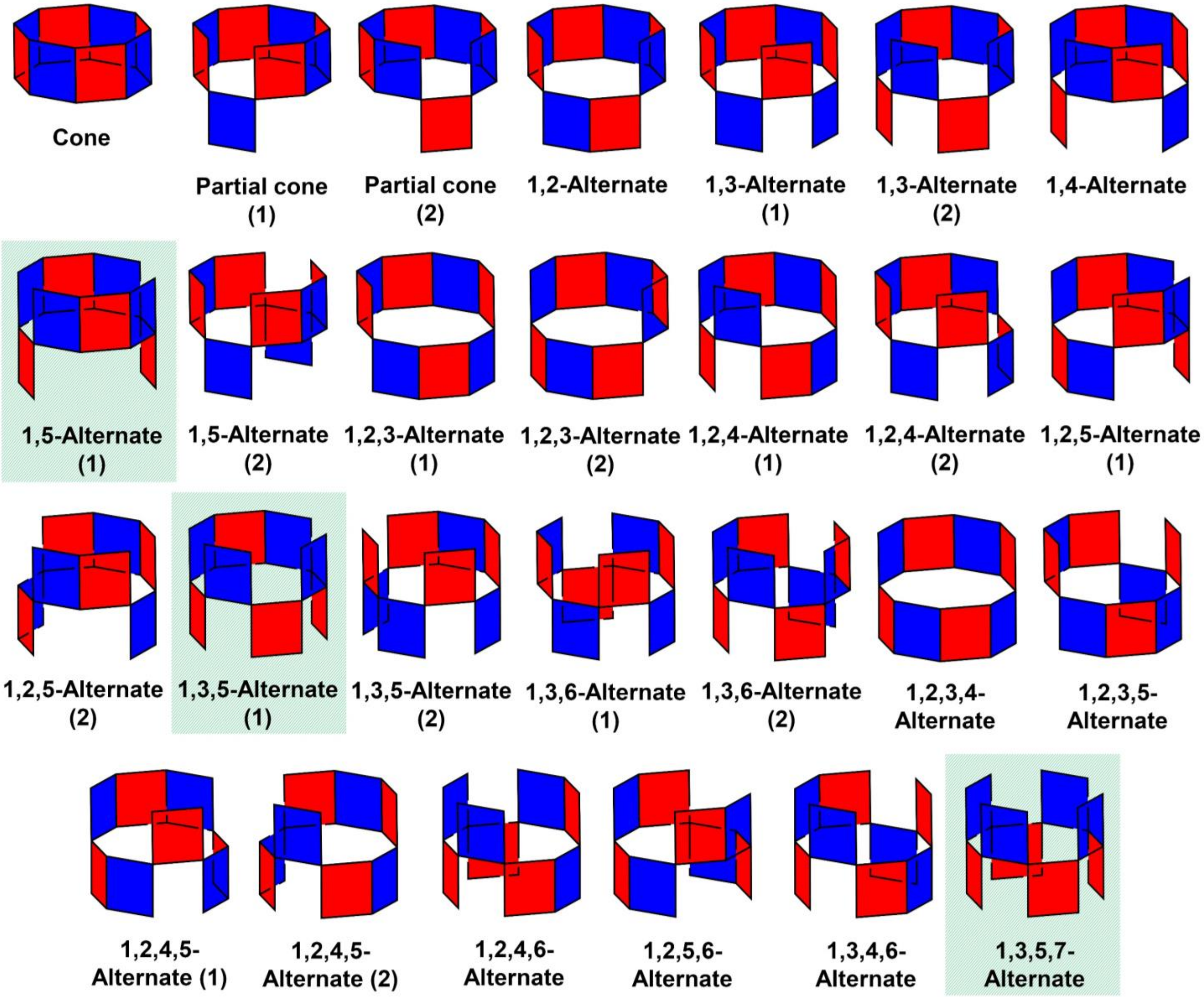

Figure S14: Schematization of the possible conformers of **C_4N_4-Me**.

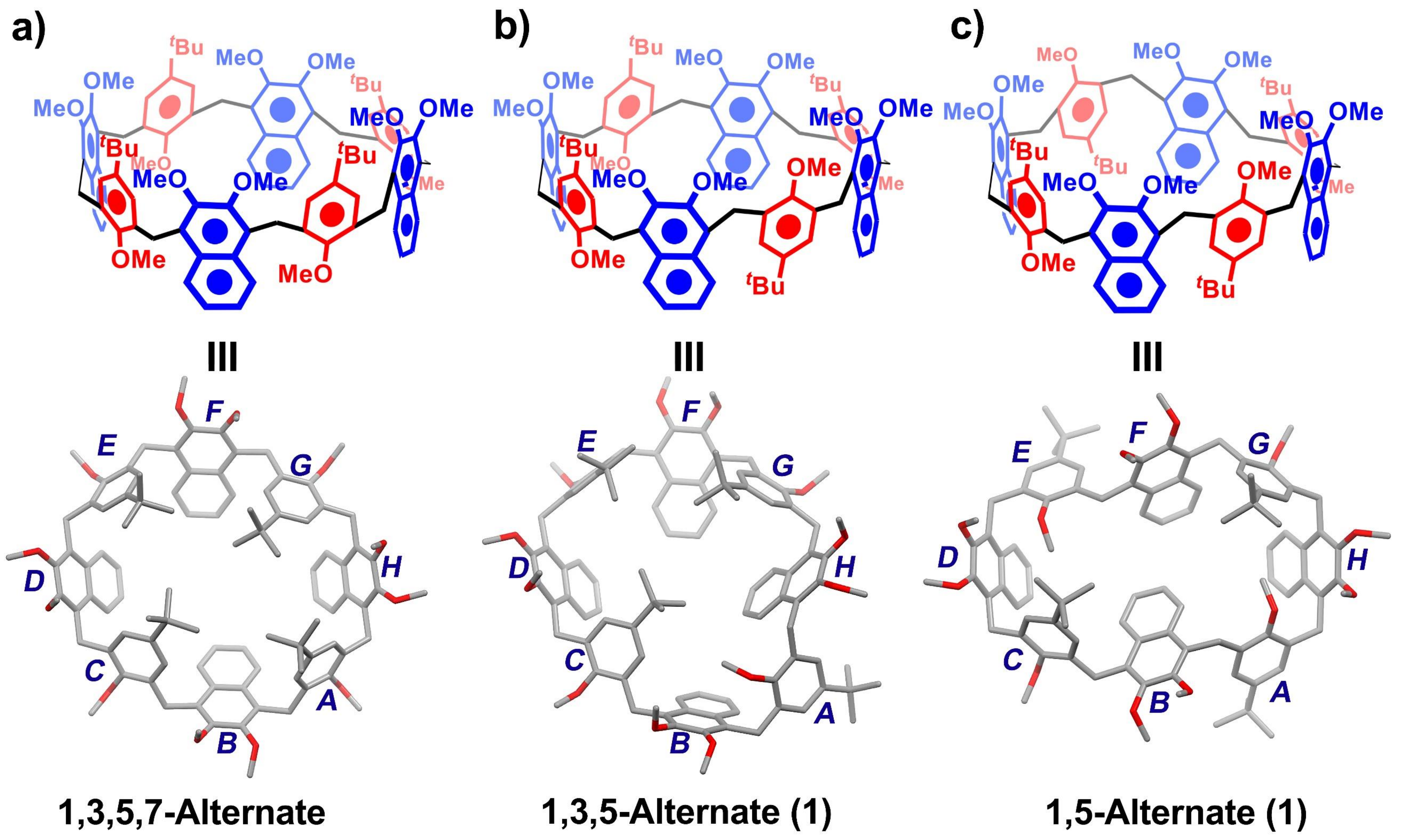

Figure S15: Structure of a) 1,3,5,7- alternate b) 1,3,5-alternate (1) and c) 1,5-alternate (1) conformation obtained by molecular dynamics calculation.

Atomic coordinates of 1,5-Alternate (I) of C_4N_4-Me

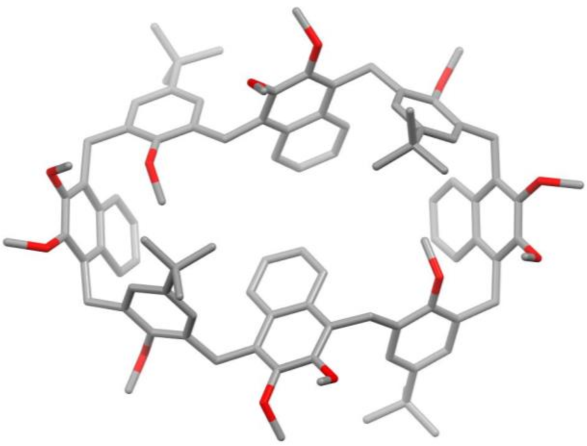

C	3.36100000	5.85100000	17.76900000
C	3.58200000	6.46500000	16.51700000
C	3.82100000	5.68200000	15.36700000
C	3.75600000	4.27300000	15.43000000
C	3.22800000	2.24900000	16.73800000
H	3.30600000	1.61900000	15.86000000
C	2.95500000	1.62100000	17.95800000
H	2.84200000	0.54100000	18.00000000
C	2.84000000	2.38400000	19.12100000
H	2.63700000	1.90100000	20.07300000
C	2.97500000	3.77700000	19.05700000
H	2.87000000	4.32400000	19.98400000
C	3.23600000	4.43600000	17.83100000
C	3.39300000	3.65300000	16.65600000
C	2.68800000	8.54600000	15.58400000
H	2.86800000	8.30500000	14.53000000
H	2.80500000	9.62800000	15.71600000
H	1.65600000	8.27200000	15.83600000
C	5.48800000	6.89100000	14.08300000
H	5.63000000	7.65000000	14.86300000
H	5.60700000	7.36700000	13.10300000
H	6.26400000	6.12500000	14.19400000
C	4.16200000	3.44000000	14.19400000
H	4.46700000	4.07800000	13.35800000
H	3.26800000	2.92000000	13.82800000
C	5.29900000	2.43100000	14.44200000
C	6.47300000	2.76200000	15.16100000
C	7.47300000	1.78300000	15.37700000
C	7.28400000	0.48300000	14.85800000
H	8.05700000	-0.25900000	15.01200000
C	6.12200000	0.13300000	14.14000000
C	5.14700000	1.12800000	13.93600000
H	4.24800000	0.88300000	13.37800000
C	5.88400000	-1.29400000	13.57600000
C	4.57900000	-1.88100000	14.18600000
H	3.69800000	-1.29100000	13.90200000
H	4.63600000	-1.89500000	15.28100000
H	4.41100000	-2.91000000	13.84300000
C	5.73600000	-1.22400000	12.03000000
H	6.62800000	-0.77200000	11.57700000
H	4.86700000	-0.62500000	11.73300000
H	5.61000000	-2.22600000	11.60000000
C	7.04000000	-2.28900000	13.89600000
H	7.17900000	-2.40000000	14.97900000
H	7.98700000	-1.95100000	13.45600000
H	6.82500000	-3.28500000	13.48800000
C	6.60800000	4.37400000	17.01800000
H	6.26800000	5.40600000	17.15400000
H	7.60200000	4.28800000	17.46800000
H	5.91800000	3.70800000	17.55000000
C	8.75800000	2.13800000	16.14500000
H	9.16000000	3.07200000	15.73600000
H	8.50900000	2.38900000	17.18100000
C	9.84400000	1.04600000	16.17900000
C	10.97200000	1.16400000	15.34000000
C	11.98900000	0.18500000	15.36300000
C	11.93900000	-0.87700000	16.29200000
C	10.73300000	-2.03900000	18.10700000
H	11.53900000	-2.75300000	18.23500000
C	9.60200000	-2.18900000	18.91900000
H	9.55200000	-3.00300000	19.63600000
C	8.53400000	-1.29800000	18.79800000
H	7.64700000	-1.42200000	19.41500000
C	8.61300000	-0.23500000	17.89000000
H	7.76000000	0.43300000	17.82400000
C	9.75500000	-0.05500000	17.07200000
C	10.81800000	-0.99300000	17.15600000
C	12.13300000	3.22400000	14.78100000
H	12.03100000	3.62800000	15.79600000
H	12.06800000	4.05300000	14.06500000
H	13.12200000	2.75800000	14.68700000
C	12.81800000	0.18400000	13.06800000
H	12.29700000	-0.75000000	12.82300000
H	13.77200000	0.20500000	12.52800000
H	12.20500000	1.02500000	12.72300000
C	13.11500000	-1.86900000	16.36300000
H	12.73100000	-2.89400000	16.40300000
H	13.71100000	-1.84800000	15.44400000
C	14.03600000	-1.55500000	17.54600000
C	14.41400000	-2.53800000	18.48400000
C	15.20900000	-2.18400000	19.59500000
C	15.66400000	-0.85700000	19.72600000
H	16.28800000	-0.61300000	20.57800000
C	15.32500000	0.13500000	18.78100000
C	14.50300000	-0.23800000	17.70100000
H	14.22000000	0.50800000	16.96600000
C	15.81200000	1.60500000	18.89300000
C	14.58400000	2.55500000	18.97900000
H	13.97200000	2.51100000	18.07000000
H	14.89900000	3.59800000	19.11400000

H	13.94200000	2.28500000	19.82600000
C	16.64400000	1.97100000	17.63100000
H	17.03900000	2.99300000	17.70100000
H	16.04000000	1.91200000	16.71700000
H	17.49400000	1.28700000	17.51700000
C	16.70800000	1.86700000	20.14000000
H	16.16400000	1.66600000	21.07100000
H	17.03800000	2.91400000	20.17300000
H	17.60800000	1.23900000	20.12000000
C	14.85400000	-4.70700000	17.52500000
H	14.95800000	-4.31300000	16.50600000
H	14.41500000	-5.71100000	17.47200000
H	15.85200000	-4.78100000	17.97700000
C	3.31700000	6.73100000	19.03400000
H	2.51100000	6.41200000	19.70300000
H	3.04600000	7.76400000	18.78700000
O	3.60800000	7.84700000	16.44300000
O	4.18200000	6.29400000	14.18400000
O	6.64300000	4.05800000	15.61200000
O	11.08900000	2.27000000	14.52100000
O	13.05500000	0.26100000	14.48600000
O	14.01600000	-3.84400000	18.30900000
C	15.13700000	-2.71200000	22.07100000
C	16.07900000	-2.09900000	22.92400000
C	15.67300000	-1.52900000	24.14900000
C	14.33000000	-1.62800000	24.57900000
C	12.05900000	-2.51300000	24.19900000
H	11.72200000	-2.16300000	25.16700000
C	11.11700000	-3.14900000	23.38100000
H	10.09200000	-3.26600000	23.72400000
C	11.49400000	-3.61700000	22.12200000
H	10.76500000	-4.10500000	21.48200000
C	12.81600000	-3.46200000	21.68700000
H	13.06000000	-3.83300000	20.69900000
C	13.79000000	-2.84200000	22.50600000
C	13.39700000	-2.33500000	23.77400000
C	18.42500000	-2.67500000	23.31600000
H	18.49900000	-2.22200000	24.31100000
H	19.39600000	-2.57700000	22.81600000
H	18.19700000	-3.74200000	23.43200000
C	17.07700000	0.43100000	24.45900000
H	17.53800000	0.33500000	23.46900000
H	17.82500000	0.82100000	25.16000000
H	16.25400000	1.15400000	24.39400000
C	13.90700000	-0.94100000	25.89400000
H	14.75700000	-0.45300000	26.38300000
H	13.61200000	-1.72700000	26.59900000
C	12.78600000	0.11200000	25.78200000
C	12.78800000	1.15100000	24.81800000
C	11.77800000	2.14300000	24.84800000
C	10.75600000	2.05600000	25.81700000
H	9.99900000	2.83100000	25.84100000
C	10.71000000	1.00600000	26.75500000
C	11.74700000	0.05600000	26.73000000
H	11.75300000	-0.73900000	27.47000000
C	9.58400000	0.88200000	27.81800000
C	8.87700000	-0.49500000	27.66700000
H	9.56500000	-1.32700000	27.85900000
H	8.47700000	-0.61500000	26.65200000
H	8.04200000	-0.58900000	28.37300000
C	10.20000000	0.98500000	29.24100000
H	10.74200000	1.93200000	29.36000000
H	10.90500000	0.16700000	29.43600000

H	9.42000000	0.94100000	30.01200000
C	8.49100000	1.98700000	27.69300000
H	8.01300000	1.96100000	26.70500000
H	8.91700000	2.98600000	27.84800000
H	7.70300000	1.84600000	28.44500000
C	13.59100000	0.72600000	22.54900000
H	14.54000000	0.38600000	22.11900000
H	13.18200000	1.50800000	21.90100000
H	12.89100000	-0.11800000	22.55300000
C	11.80700000	3.36100000	23.90800000
H	12.45700000	4.11700000	24.36000000
H	12.30500000	3.13400000	22.96200000
C	10.40500000	3.93600000	23.61200000
C	9.98900000	5.11500000	24.26900000
C	8.67800000	5.61000000	24.09300000
C	7.79200000	4.98900000	23.18700000
C	7.36300000	3.18800000	21.55400000
H	6.37400000	3.57600000	21.33300000
C	7.77000000	2.02900000	20.88000000
H	7.10600000	1.55500000	20.16200000
C	9.02900000	1.48100000	21.13700000
H	9.34900000	0.57600000	20.62800000
C	9.88700000	2.10000000	22.05300000
H	10.84600000	1.63200000	22.23400000
C	9.51000000	3.28900000	22.72000000
C	8.21800000	3.83000000	22.48500000
C	11.29500000	7.11200000	24.76900000
H	11.75500000	7.13200000	23.77400000
H	12.03100000	7.46300000	25.50300000
H	10.44300000	7.80100000	24.77500000
C	8.13000000	6.56800000	26.26100000
H	7.45600000	5.74100000	26.51800000
H	7.72600000	7.49500000	26.68700000
H	9.10900000	6.37400000	26.71500000
C	6.37800000	5.57200000	22.98900000
H	5.65000000	4.77100000	23.16300000
H	6.14100000	6.32600000	23.74700000
C	6.15800000	6.19600000	21.60600000
C	4.86900000	6.23500000	21.03600000
C	4.68400000	6.74300000	19.73300000
C	5.79100000	7.26200000	19.03500000
H	5.62200000	7.66600000	18.04400000
C	7.08800000	7.25600000	19.59200000
C	7.24700000	6.72400000	20.88700000
H	8.23200000	6.72100000	21.34100000
C	8.32600000	7.81200000	18.83800000
C	9.44900000	6.73500000	18.78200000
H	9.08100000	5.81300000	18.31500000
H	9.82200000	6.47900000	19.78000000
H	10.30600000	7.08900000	18.19500000
C	8.86400000	9.05600000	19.59800000
H	9.72700000	9.49300000	19.08000000
H	9.18100000	8.79800000	20.61600000
H	8.08800000	9.82800000	19.67400000
C	8.01500000	8.24100000	17.37300000
H	7.62100000	7.40000000	16.78900000
H	8.92100000	8.59900000	16.86800000
H	7.28100000	9.05700000	17.34500000
C	3.11600000	6.72600000	22.62000000
H	3.80900000	7.12100000	23.37400000
H	2.28100000	6.23200000	23.13300000
H	2.72300000	7.56300000	22.03100000
C	15.58900000	-3.19600000	20.68000000

H	15.18300000	-4.19300000	20.47800000
H	16.67400000	-3.35000000	20.64600000
O	17.40300000	-2.03600000	22.52700000
O	16.58700000	-0.84100000	24.92200000
O	13.80500000	1.22000000	23.88900000
O	10.88000000	5.77300000	25.09500000
O	8.23700000	6.69200000	24.83100000
O	3.79100000	5.79000000	21.76500000

1 2 1.5 13 1.5 104 1.0
2 3 1.5 107 1.0
3 4 1.5 108 1.0
4 14 1.5 23 1.0
5 6 1.0 7 1.5 14 1.5
6
7 8 1.0 9 1.5
8
9 10 1.0 11 1.5
10
11 12 1.0 13 1.5
12
13 14 1.5
14
15 16 1.0 17 1.0 18 1.0 107 1.0
16
17
18
19 20 1.0 21 1.0 22 1.0 108 1.0
20
21
22
23 24 1.0 25 1.0 26 1.0
24
25
26 27 1.5 32 1.5
27 28 1.5 109 1.0
28 29 1.5 51 1.0
29 30 1.0 31 1.5
30
31 32 1.5 34 1.0
32 33 1.0
33
34 35 1.0 39 1.0 43 1.0
35 36 1.0 37 1.0 38 1.0
36
37
38
39 40 1.0 41 1.0 42 1.0
40
41
42
43 44 1.0 45 1.0 46 1.0
44
45
46
47 48 1.0 49 1.0 50 1.0 109 1.0
48
49
50
51 52 1.0 53 1.0 54 1.0
52
53
54 55 2.0 66 1.0
55 56 1.0 110 1.0
56 57 2.0 111 1.0
57 67 1.0 76 1.0
58 59 1.0 60 1.5 67 1.5
59
60 61 1.0 62 1.5
61
62 63 1.0 64 1.5
63
64 65 1.0 66 1.5
65
66 67 1.5
67
68 69 1.0 70 1.0 71 1.0 110 1.0
69
70
71
72 73 1.0 74 1.0 75 1.0 111 1.0
73
74
75
76 77 1.0 78 1.0 79 1.0
77
78
79 80 1.5 85 1.5
80 81 1.5 112 1.0
81 82 1.5 216 1.0
82 83 1.0 84 1.5
83
84 85 1.5 87 1.0
85 86 1.0
86
87 88 1.0 92 1.0 96 1.0
88 89 1.0 90 1.0 91 1.0
89
90
91
92 93 1.0 94 1.0 95 1.0
93
94
95
96 97 1.0 98 1.0 99 1.0
97
98
99
100 101 1.0 102 1.0 103 1.0 112 1.0
101
102
103
104 105 1.0 106 1.0 193 1.0
105
106
107
108
109
110
111
112
113 114 2.0 125 1.0 216 1.0
114 115 1.0 219 1.0
115 116 2.0 220 1.0
116 126 1.0 135 1.0
117 118 1.0 119 1.5 126 1.5

118
119 120 1.0 121 1.5
120
121 122 1.0 123 1.5
122
123 124 1.0 125 1.5
124
125 126 1.5
126
127 128 1.0 129 1.0 130 1.0 219 1.0
128
129
130
131 132 1.0 133 1.0 134 1.0 220 1.0
132
133
134
135 136 1.0 137 1.0 138 1.0
136
137
138 139 1.5 144 1.5
139 140 1.5 221 1.0
140 141 1.5 163 1.0
141 142 1.0 143 1.5
142
143 144 1.5 146 1.0
144 145 1.0
145
146 147 1.0 151 1.0 155 1.0
147 148 1.0 149 1.0 150 1.0
148
149
150
151 152 1.0 153 1.0 154 1.0
152
153
154
155 156 1.0 157 1.0 158 1.0
156
157
158
159 160 1.0 161 1.0 162 1.0 221 1.0
160
161
162
163 164 1.0 165 1.0 166 1.0
164
165
166 167 1.5 178 1.5
167 168 1.5 222 1.0
168 169 1.5 223 1.0
169 179 1.5 188 1.0
170 171 1.0 172 1.5 179 1.5
171
172 173 1.0 174 1.5
173
174 175 1.0 176 1.5
175
176 177 1.0 178 1.5
177
178 179 1.5
179
180 181 1.0 182 1.0 183 1.0 222 1.0
181
182
183
184 185 1.0 186 1.0 187 1.0 223 1.0
185
186
187
188 189 1.0 190 1.0 191 1.0
189
190
191 192 1.5 197 1.5
192 193 1.5 224 1.0
193 194 1.5
194 195 1.0 196 1.5
195
196 197 1.5 199 1.0
197 198 1.0
198
199 200 1.0 204 1.0 208 1.0
200 201 1.0 202 1.0 203 1.0
201
202
203
204 205 1.0 206 1.0 207 1.0
205
206
207
208 209 1.0 210 1.0 211 1.0
209
210
211
212 213 1.0 214 1.0 215 1.0 224 1.0
213
214
215
216 217 1.0 218 1.0
217
218
219
220
221
222
223
224

Atomic coordinates of 1,3,5-Alternate (I) of C_4N_4-Me

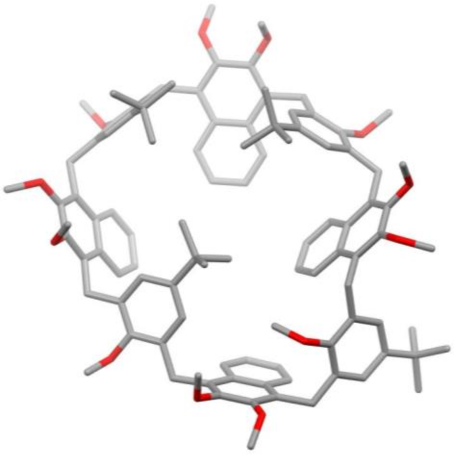

C	8.28900000	3.92700000	13.47600000
C	9.34500000	4.12300000	12.55900000
C	10.50400000	3.31600000	12.61900000
C	10.59500000	2.25700000	13.54700000
C	9.62000000	1.03300000	15.45300000
H	10.48300000	0.38000000	15.51700000
C	8.59900000	0.85200000	16.39400000
H	8.68600000	0.07700000	17.14900000
C	7.47600000	1.68100000	16.37100000
H	6.69200000	1.55800000	17.11300000
C	7.36900000	2.68600000	15.40200000
H	6.48400000	3.31000000	15.42700000
C	8.39400000	2.89300000	14.44500000
C	9.53800000	2.05300000	14.47300000
C	9.26900000	4.78000000	10.20300000
H	10.24500000	4.36700000	9.92200000
H	9.08800000	5.67900000	9.60100000
H	8.49400000	4.04000000	9.97100000
C	12.33600000	4.79000000	12.01800000
H	11.69300000	5.67400000	11.92900000
H	13.14400000	4.86700000	11.28100000
H	12.77900000	4.78100000	13.02200000
C	11.85200000	1.36800000	13.56100000
H	12.45900000	1.51600000	12.66100000
H	11.52900000	0.32200000	13.49700000
C	12.74600000	1.58200000	14.79000000
C	13.47900000	0.51100000	15.33800000
C	14.25600000	0.70000000	16.49900000
C	14.33800000	1.98400000	17.07200000
H	14.93500000	2.11000000	17.96700000
C	13.62500000	3.07600000	16.53500000
C	12.82200000	2.84900000	15.39800000
H	12.22900000	3.66300000	14.99800000
C	13.64100000	4.48000000	17.19600000
C	13.92000000	5.58100000	16.13000000
H	13.11300000	5.65200000	15.39200000
H	14.85300000	5.37100000	15.59200000
H	14.01500000	6.56800000	16.59900000
C	12.25400000	4.73600000	17.84200000
H	12.04100000	3.98600000	18.61600000
H	11.44900000	4.68100000	17.09900000
H	12.21400000	5.72700000	18.31100000
C	14.72100000	4.62500000	18.30900000
H	15.72200000	4.39500000	17.92000000
H	14.51500000	3.96000000	19.15700000
H	14.74500000	5.65000000	18.70200000
C	14.43300000	-1.10200000	13.81400000
H	14.44100000	-0.40300000	12.96900000
H	14.24400000	-2.11300000	13.43400000
H	15.41800000	-1.08400000	14.29700000
C	14.95000000	-0.48200000	17.18900000
H	14.48100000	-1.43200000	16.90800000
H	15.97500000	-0.55600000	16.80900000
C	14.91100000	-0.35800000	18.72500000
C	16.11000000	-0.15600000	19.43800000
C	16.09200000	0.01000000	20.84000000
C	14.87100000	0.05400000	21.54600000
C	12.40200000	-0.10200000	21.49100000
H	12.32900000	0.08500000	22.55200000
C	11.20400000	-0.31300000	20.79900000
H	10.25700000	-0.28400000	21.33100000
C	11.23000000	-0.55100000	19.42500000
H	10.30300000	-0.70600000	18.88200000
C	12.45400000	-0.57700000	18.74600000
H	12.42800000	-0.76400000	17.67800000
C	13.67800000	-0.36500000	19.42900000
C	13.65400000	-0.13400000	20.83000000
C	18.02900000	1.15200000	18.71400000
H	17.40500000	1.94200000	18.27600000
H	18.93400000	1.04200000	18.10300000
H	18.32600000	1.46000000	19.72400000
C	18.13100000	-1.03600000	21.63500000
H	17.59600000	-1.85500000	22.13200000
H	19.02500000	-0.79300000	22.22200000
H	18.44600000	-1.37400000	20.64000000
C	14.89100000	0.28500000	23.07500000
H	15.91100000	0.41200000	23.45400000
H	14.41700000	1.24800000	23.27700000
C	14.24500000	-0.85200000	23.87300000
C	13.41100000	-0.61600000	24.99200000
C	12.82100000	-1.70900000	25.66800000
C	13.10100000	-3.02300000	25.23900000
H	12.65600000	-3.84600000	25.78700000
C	13.92400000	-3.27700000	24.12200000
C	14.48800000	-2.17400000	23.45800000
H	15.12400000	-2.34300000	22.59500000
C	14.21400000	-4.70800000	23.59600000
C	15.74600000	-4.97400000	23.62300000
H	16.14300000	-4.84300000	24.63800000
H	16.28800000	-4.29100000	22.95800000
H	15.97400000	-5.99900000	23.30100000
C	13.70400000	-4.83000000	22.13200000

H	13.85200000	-5.84600000	21.74500000
H	14.23100000	-4.14100000	21.46000000
H	12.63200000	-4.59900000	22.07500000
C	13.52300000	-5.82500000	24.43200000
H	13.86500000	-5.80800000	25.47500000
H	13.75300000	-6.81800000	24.02700000
H	12.43100000	-5.71000000	24.42200000
C	12.22600000	1.53900000	24.88200000
H	12.57900000	1.97400000	23.94200000
H	12.00300000	2.36000000	25.57400000
H	11.29700000	0.99500000	24.67600000
C	7.05100000	4.84400000	13.41600000
H	6.16100000	4.21000000	13.33100000
H	7.03900000	5.45500000	12.50700000
O	9.22900000	5.11500000	11.60200000
O	11.58100000	3.58400000	11.79800000
O	13.41100000	-0.73300000	14.75200000
O	17.30500000	-0.09100000	18.74700000
O	17.28100000	0.12000000	21.53400000
O	13.21300000	0.66600000	25.46700000
C	10.67200000	-0.59900000	26.66000000
C	10.51000000	0.53900000	27.48500000
C	9.43900000	1.43500000	27.27400000
C	8.47400000	1.18300000	26.28000000
C	7.70000000	-0.20100000	24.39900000
H	6.88300000	0.48200000	24.19400000
C	7.82900000	-1.32600000	23.57500000
H	7.11800000	-1.49600000	22.77100000
C	8.86900000	-2.23000000	23.79300000
H	8.96700000	-3.11200000	23.16500000
C	9.79100000	-2.00100000	24.82200000
H	10.56900000	-2.73900000	24.96400000
C	9.70200000	-0.85600000	25.64900000
C	8.62000000	0.04400000	25.44800000
C	10.96700000	0.74400000	29.87500000
H	10.22400000	1.52900000	30.06400000
H	11.81900000	0.90300000	30.54700000
H	10.51600000	-0.23000000	30.10400000
C	10.31200000	3.63700000	27.79800000
H	11.32800000	3.27500000	27.99600000
H	10.10500000	4.48500000	28.46200000
H	10.25800000	3.98500000	26.75800000
C	7.27800000	2.14300000	26.13200000
H	7.15300000	2.74700000	27.03700000
H	6.35700000	1.54900000	26.09000000
C	7.33600000	3.08700000	24.92300000
C	6.17800000	3.79000000	24.53700000
C	6.19500000	4.64600000	23.41700000
C	7.39400000	4.81000000	22.69600000
H	7.39000000	5.46400000	21.83200000
C	8.57700000	4.13400000	23.07400000
C	8.52500000	3.27500000	24.19300000
H	9.42100000	2.74300000	24.49000000
C	9.91300000	4.29300000	22.29800000
C	11.00800000	4.83900000	23.25700000
H	11.18700000	4.16100000	24.09900000
H	10.71000000	5.81200000	23.66900000
H	11.96200000	4.97300000	22.73000000
C	10.35900000	2.91100000	21.74500000
H	9.59900000	2.50200000	21.06700000
H	10.51500000	2.18200000	22.54800000
H	11.30000000	2.99300000	21.18400000
C	9.81500000	5.27200000	21.09100000
H	9.52400000	6.27800000	21.41700000
H	9.08300000	4.91900000	20.35300000
H	10.78200000	5.35900000	20.57900000
C	4.72000000	4.55900000	26.31400000
H	4.63200000	5.57600000	25.91300000
H	5.51600000	4.54700000	27.07000000
H	3.77300000	4.28800000	26.79600000
C	4.91900000	5.38800000	22.99500000
H	4.03500000	4.79000000	23.24600000
H	4.81900000	6.28400000	23.61800000
C	4.83900000	5.74800000	21.50000000
C	4.97400000	7.09600000	21.10500000
C	4.79800000	7.47100000	19.75400000
C	4.58100000	6.49000000	18.76200000
C	4.35200000	4.10300000	18.17400000
H	4.28900000	4.33500000	17.11800000
C	4.27200000	2.75500000	18.54600000
H	4.14000000	1.99000000	17.78500000
C	4.37000000	2.39600000	19.89100000
H	4.31600000	1.35000000	20.18200000
C	4.55100000	3.38500000	20.86500000
H	4.64300000	3.06500000	21.89800000
C	4.63300000	4.75400000	20.50900000
C	4.52400000	5.12100000	19.14200000
C	6.61800000	8.65600000	21.95900000
H	7.40300000	7.89300000	22.02900000
H	6.75200000	9.37300000	22.77900000
H	6.73300000	9.18800000	21.00700000
C	3.88600000	9.73600000	19.94500000
H	2.86100000	9.39400000	19.75400000
H	4.01900000	10.72200000	19.48300000
H	4.02600000	9.83900000	21.02700000
C	4.40800000	6.93100000	17.29400000
H	3.56400000	6.39400000	16.84800000
H	4.10500000	7.98200000	17.23200000
C	5.69200000	6.75300000	16.47900000
C	5.73300000	5.97400000	15.30500000
C	6.95300000	5.78000000	14.62500000
C	8.11300000	6.43000000	15.08600000
H	9.03700000	6.27300000	14.54000000
C	8.08800000	7.24900000	16.23600000
C	6.86600000	7.38600000	16.92300000
H	6.82500000	7.99100000	17.82200000
C	9.35100000	7.96400000	16.78500000
C	9.64600000	7.44700000	18.22000000
H	9.74000000	6.35400000	18.22200000
H	8.84400000	7.71400000	18.92000000
H	10.58100000	7.87000000	18.61100000
C	9.10200000	9.49800000	16.83900000
H	9.99700000	10.03000000	17.18400000
H	8.28300000	9.75100000	17.52400000
H	8.84000000	9.88300000	15.84500000
C	10.62100000	7.72300000	15.91600000
H	10.86200000	6.65400000	15.85900000
H	11.49300000	8.23400000	16.34300000
H	10.48000000	8.10100000	14.89500000
C	3.84200000	6.09800000	13.81000000
H	3.52100000	7.07800000	14.18500000
H	2.95400000	5.52000000	13.52600000
H	4.46600000	6.24700000	12.92000000
C	11.89700000	-1.52100000	26.88400000
H	11.52600000	-2.49400000	27.22800000
H	12.52300000	-1.16500000	27.70800000

O 11.41100000 0.77200000 28.50700000
O 9.34800000 2.59400000 28.01900000
O 5.01800000 3.62800000 25.26100000
O 5.31900000 8.04100000 22.05000000
O 4.82900000 8.80400000 19.38500000
O 4.57900000 5.39700000 14.82400000

1 2 1.5 13 1.5 104 1.0
2 3 1.5 107 1.0
3 4 1.5 108 1.0
4 14 1.5 23 1.0
5 6 1.0 7 1.5 14 1.5
6
7 8 1.0 9 1.5
8
9 10 1.0 11 1.5
10
11 12 1.0 13 1.5
12
13 14 1.5
14
15 16 1.0 17 1.0 18 1.0 107 1.0
16
17
18
19 20 1.0 21 1.0 22 1.0 108 1.0
20
21
22
23 24 1.0 25 1.0 26 1.0
24
25
26 27 1.5 32 1.5
27 28 1.5 109 1.0
28 29 1.5 51 1.0
29 30 1.0 31 1.5
30
31 32 1.5 34 1.0
32 33 1.0
33
34 35 1.0 39 1.0 43 1.0
35 36 1.0 37 1.0 38 1.0
36
37
38
39 40 1.0 41 1.0 42 1.0
40
41
42
43 44 1.0 45 1.0 46 1.0
44
45
46
47 48 1.0 49 1.0 50 1.0 109 1.0
48
49
50
51 52 1.0 53 1.0 54 1.0
52
53
54 55 1.5 66 1.5
55 56 1.5 110 1.0
56 57 1.5 111 1.0
57 67 1.5 76 1.0
58 59 1.0 60 1.5 67 1.5
59
60 61 1.0 62 1.5
61
62 63 1.0 64 1.5
63
64 65 1.0 66 1.5
65
66 67 1.5
67
68 69 1.0 70 1.0 71 1.0 110 1.0
69
70
71
72 73 1.0 74 1.0 75 1.0 111 1.0
73
74
75
76 77 1.0 78 1.0 79 1.0
77
78
79 80 1.5 85 1.5
80 81 1.5 112 1.0
81 82 1.5 216 1.0
82 83 1.0 84 1.5
83
84 85 1.5 87 1.0
85 86 1.0
86
87 88 1.0 92 1.0 96 1.0
88 89 1.0 90 1.0 91 1.0
89
90
91
92 93 1.0 94 1.0 95 1.0
93
94
95
96 97 1.0 98 1.0 99 1.0
97
98
99
100 101 1.0 102 1.0 103 1.0 112 1.0
101
102
103
104 105 1.0 106 1.0 193 1.0
105
106
107
108
109
110
111
112
113 114 1.5 125 1.5 216 1.0
114 115 1.5 219 1.0
115 116 1.5 220 1.0
116 126 1.5 135 1.0
117 118 1.0 119 2.0 126 1.0
118
119 120 1.0 121 1.0

120
121 122 1.0 123 2.0
122
123 124 1.0 125 1.0
124
125 126 1.5
126
127 128 1.0 129 1.0 130 1.0 219 1.0
128
129
130
131 132 1.0 133 1.0 134 1.0 220 1.0
132
133
134
135 136 1.0 137 1.0 138 1.0
136
137
138 139 1.5 144 1.5
139 140 1.5 221 1.0
140 141 1.5 163 1.0
141 142 1.0 143 1.5
142
143 144 1.5 146 1.0
144 145 1.0
145
146 147 1.0 151 1.0 155 1.0
147 148 1.0 149 1.0 150 1.0
148
149
150
151 152 1.0 153 1.0 154 1.0
152
153
154
155 156 1.0 157 1.0 158 1.0
156
157
158
159 160 1.0 161 1.0 162 1.0 221 1.0
160
161
162
163 164 1.0 165 1.0 166 1.0
164
165
166 167 1.5 178 1.5
167 168 1.5 222 1.0
168 169 1.5 223 1.0
169 179 1.5 188 1.0
170 171 1.0 172 2.0 179 1.0
171
172 173 1.0 174 1.0
173
174 175 1.0 176 2.0
175
176 177 1.0 178 1.0
177
178 179 1.5
179
180 181 1.0 182 1.0 183 1.0 222 1.0
181
182
183
184 185 1.0 186 1.0 187 1.0 223 1.0
185
186
187
188 189 1.0 190 1.0 191 1.0
189
190
191 192 1.5 197 1.5
192 193 1.5 224 1.0
193 194 1.5
194 195 1.0 196 1.5
195
196 197 1.5 199 1.0
197 198 1.0
198
199 200 1.0 204 1.0 208 1.0
200 201 1.0 202 1.0 203 1.0
201
202
203
204 205 1.0 206 1.0 207 1.0
205
206
207
208 209 1.0 210 1.0 211 1.0
209
210
211
212 213 1.0 214 1.0 215 1.0 224 1.0
213
214
215
216 217 1.0 218 1.0
217
218
219
220
221
222
223
224

Atomic coordinates of 1,3,5,7-Alternate of C_4N_4-Me

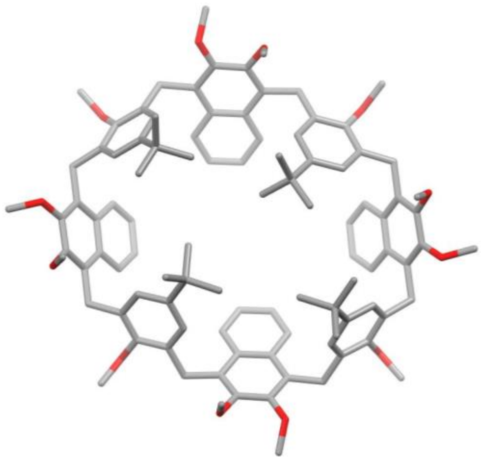

C	13.75900000	3.83600000	14.49200000
C	15.12400000	3.61500000	14.77800000
C	15.52200000	2.48800000	15.53200000
C	14.57500000	1.52900000	15.94600000
C	12.21100000	0.87500000	16.18900000
H	12.48200000	-0.01200000	16.75200000
C	10.84800000	1.12800000	15.99000000
H	10.10500000	0.44400000	16.39100000
C	10.44600000	2.27100000	15.29700000
H	9.38900000	2.48200000	15.15900000
C	11.40600000	3.15900000	14.79500000
H	11.05100000	4.04200000	14.27500000
C	12.78900000	2.92800000	14.99400000
C	13.19700000	1.77000000	15.70700000
C	17.13500000	4.07500000	13.44800000
H	17.82100000	3.40800000	13.98200000
H	17.70500000	4.94200000	13.09300000
H	16.72900000	3.54200000	12.57900000
C	17.35700000	3.21200000	16.92400000
H	18.41000000	2.97900000	17.12100000
H	16.78900000	3.08900000	17.85500000
H	17.28200000	4.25900000	16.60400000
C	15.05800000	0.24300000	16.64400000
H	16.13700000	0.10500000	16.51100000
H	14.61600000	-0.61000000	16.11600000
C	14.73900000	0.14600000	18.14100000
C	14.70200000	-1.11700000	18.76500000
C	14.36100000	-1.23300000	20.12700000
C	14.09400000	-0.06800000	20.86400000
H	13.81900000	-0.15900000	21.90900000
C	14.15000000	1.21100000	20.27600000
C	14.47100000	1.30100000	18.90300000
H	14.50200000	2.26600000	18.41000000
C	13.83100000	2.45500000	21.14700000
C	14.79800000	2.50900000	22.36300000
H	15.84300000	2.50800000	22.02700000
H	14.65600000	1.65300000	23.03300000
H	14.63400000	3.41800000	22.95600000
C	13.96400000	3.79800000	20.37400000
H	13.26200000	3.83700000	19.53200000
H	14.98200000	3.93500000	19.98800000
H	13.73900000	4.65100000	21.02700000
C	12.37100000	2.35700000	21.66700000
H	12.20900000	1.44800000	22.26000000
H	11.65900000	2.34000000	20.83200000
H	12.12700000	3.21600000	22.30500000
C	16.33200000	-2.75100000	18.04500000
H	17.02600000	-1.99300000	17.66100000
H	16.40200000	-3.64600000	17.41500000
H	16.62800000	-3.01400000	19.06800000
C	14.25300000	-2.60200000	20.81000000
H	14.16200000	-3.40600000	20.07000000
H	15.20000000	-2.81200000	21.31900000
C	13.05700000	-2.69900000	21.77600000
C	13.28800000	-2.75700000	23.16700000
C	12.20600000	-2.87700000	24.06900000
C	10.87500000	-2.85300000	23.59900000
C	9.31200000	-2.70500000	21.69400000
H	8.45300000	-2.72100000	22.35500000
C	9.06700000	-2.62300000	20.31800000
H	8.04600000	-2.58700000	19.95000000
C	10.13700000	-2.57700000	19.42200000
H	9.95200000	-2.50400000	18.35400000
C	11.45200000	-2.61100000	19.90400000
H	12.25300000	-2.56000000	19.17500000
C	11.72100000	-2.69400000	21.29200000
C	10.63300000	-2.75100000	22.20200000
C	14.96500000	-1.54800000	24.46300000
H	14.38800000	-1.52900000	25.39400000
H	14.79300000	-0.60600000	23.92800000
H	16.03000000	-1.61500000	24.71500000
C	13.13600000	-4.19200000	25.90300000
H	12.60800000	-5.10300000	25.59400000
H	13.18900000	-4.17000000	26.99800000
H	14.15700000	-4.22700000	25.50600000
C	9.71400000	-2.93200000	24.60800000
H	9.00500000	-3.69800000	24.27700000
H	10.05400000	-3.29400000	25.58500000
C	9.03200000	-1.57500000	24.81200000
C	7.63300000	-1.42500000	24.71800000
C	7.04300000	-0.15400000	24.86800000
C	7.86000000	0.95400000	25.15600000
H	7.39800000	1.92800000	25.28100000
C	9.25900000	0.83000000	25.27500000
C	9.82900000	-0.45000000	25.09600000
H	10.90200000	-0.59200000	25.17000000
C	10.11000000	2.09300000	25.56900000
C	11.64100000	1.80900000	25.61500000
H	11.99700000	1.39600000	24.66300000
H	11.88900000	1.10000000	26.41500000

H	12.20600000	2.73100000	25.80500000
C	9.69500000	2.69000000	26.94200000
H	10.30300000	3.57100000	27.18900000
H	9.83000000	1.95100000	27.74200000
H	8.64400000	3.00200000	26.94600000
C	9.85600000	3.15200000	24.46000000
H	10.02900000	2.71700000	23.46800000
H	10.52200000	4.01700000	24.57300000
H	8.82400000	3.52500000	24.48400000
C	6.30600000	-3.23200000	25.61100000
H	7.12000000	-3.61100000	26.24200000
H	5.69800000	-4.07900000	25.27100000
H	5.67600000	-2.56500000	26.21400000
C	13.35300000	5.03800000	13.61600000
H	12.70500000	4.66800000	12.81400000
H	14.21500000	5.45800000	13.08600000
O	16.06300000	4.51500000	14.30300000
O	16.84000000	2.33400000	15.90900000
O	14.98800000	-2.24500000	18.02900000
O	14.58500000	-2.66600000	23.63700000
O	12.43500000	-3.03000000	25.42400000
O	6.84000000	-2.52600000	24.48100000
C	5.16900000	1.31300000	23.91400000
C	4.64800000	2.43700000	24.59000000
C	4.29600000	3.60100000	23.87100000
C	4.38700000	3.63300000	22.46400000
C	5.12900000	2.55300000	20.37700000
H	4.84700000	3.42100000	19.79000000
C	5.71900000	1.47700000	19.70300000
H	5.87400000	1.52800000	18.62900000
C	6.12700000	0.34800000	20.41600000
H	6.60300000	-0.48000000	19.89900000
C	5.93800000	0.29000000	21.80300000
H	6.28400000	-0.59800000	22.32100000
C	5.34800000	1.36900000	22.50500000
C	4.94200000	2.52100000	21.78000000
C	3.22500000	2.64000000	26.58400000
H	2.93000000	3.68900000	26.46500000
H	3.28300000	2.42000000	27.65700000
H	2.45200000	2.00300000	26.13600000
C	4.94200000	5.49300000	25.21800000
H	5.44000000	4.87100000	25.97200000
H	4.50500000	6.36700000	25.71400000
H	5.69500000	5.83700000	24.49600000
C	3.89900000	4.88200000	21.70600000
H	3.27900000	5.51500000	22.34900000
H	3.21100000	4.55400000	20.91700000
C	5.00800000	5.74700000	21.09400000
C	4.69200000	6.66600000	20.07500000
C	5.70300000	7.43000000	19.46000000
C	7.03300000	7.29100000	19.90000000
H	7.80000000	7.87700000	19.40800000
C	7.37500000	6.39600000	20.93700000
C	6.34500000	5.62800000	21.51900000
H	6.59600000	4.91700000	22.30000000
C	8.83600000	6.20400000	21.42500000
C	8.92100000	6.49100000	22.95100000
H	8.30300000	5.79500000	23.53100000
H	8.57900000	7.51000000	23.17400000
H	9.95300000	6.39300000	23.31300000
C	9.27500000	4.73900000	21.15500000
H	9.19600000	4.50000000	20.08700000
H	8.65600000	4.02000000	21.70500000
H	10.31700000	4.58100000	21.45800000
C	9.85500000	7.14100000	20.71100000
H	9.60400000	8.19700000	20.87200000
H	9.88000000	6.94800000	19.63100000
H	10.87100000	6.98000000	21.09400000
C	2.58800000	7.84600000	20.25100000
H	1.57500000	7.82300000	19.83200000
H	3.03400000	8.82600000	20.04200000
H	2.52300000	7.71300000	21.33800000
C	5.38100000	8.38300000	18.30100000
H	4.42700000	8.11800000	17.83100000
H	5.20800000	9.38400000	18.71200000
C	6.46400000	8.39800000	17.20700000
C	7.26200000	9.55000000	17.03400000
C	8.23900000	9.59900000	16.01500000
C	8.50100000	8.46300000	15.21900000
C	7.98900000	6.11300000	14.66200000
H	8.78600000	6.07800000	13.92900000
C	7.22400000	4.95400000	14.84100000
H	7.43500000	4.06800000	14.24800000
C	6.19500000	4.93800000	15.78400000
H	5.60300000	4.03900000	15.93000000
C	5.93800000	6.07700000	16.55500000
H	5.14100000	6.01700000	17.28900000
C	6.70300000	7.25900000	16.39300000
C	7.73700000	7.28200000	15.42000000
C	8.18700000	10.95600000	18.79000000
H	8.42800000	10.10600000	19.44000000
H	7.89900000	11.80700000	19.41900000
H	9.08800000	11.22800000	18.22700000
C	8.27100000	11.96500000	15.39900000
H	7.69000000	11.79100000	14.48500000
H	9.00000000	12.76100000	15.20600000
H	7.59000000	12.30300000	16.18900000
C	9.62100000	8.52500000	14.16400000
H	9.25700000	8.10500000	13.22000000
H	9.87100000	9.56100000	13.90800000
C	10.89700000	7.82800000	14.64700000
C	11.53000000	6.82100000	13.89100000
C	12.67400000	6.16700000	14.39600000
C	13.20300000	6.56600000	15.63700000
H	14.09200000	6.06800000	16.01200000
C	12.60600000	7.59200000	16.39900000
C	11.44500000	8.20800000	15.88500000
H	10.94400000	8.99200000	16.44200000
C	13.22900000	7.99300000	17.76200000
C	12.43900000	9.11800000	18.49600000
H	11.40600000	8.80800000	18.69900000
H	12.41600000	10.03900000	17.90100000
H	12.90500000	9.36000000	19.46000000
C	14.68100000	8.50100000	17.53800000
H	15.13600000	8.82500000	18.48300000
H	14.69000000	9.35500000	16.84800000
H	15.32200000	7.71900000	17.11300000
C	13.26700000	6.75400000	18.69700000
H	12.26400000	6.32300000	18.81100000
H	13.63600000	7.02100000	19.69600000
H	13.92500000	5.97100000	18.30100000
C	11.55900000	7.16000000	11.50200000
H	11.37400000	8.23900000	11.58400000
H	11.07400000	6.78600000	10.59200000
H	12.64000000	6.99300000	11.42100000
C	5.53200000	0.04800000	24.71500000

H	5.06500000	-0.81500000	24.22600000
H	5.08400000	0.06300000	25.71400000
O	4.49900000	2.38100000	25.96600000
O	3.91700000	4.74300000	24.54300000
O	3.38300000	6.80000000	19.66900000
O	7.10500000	10.61300000	17.90200000
O	8.95600000	10.75900000	15.78500000
O	11.03600000	6.47900000	12.65200000

1 2 2.0 13 1.0 104 1.0
2 3 1.0 107 1.0
3 4 2.0 108 1.0
4 14 1.0 23 1.0
5 6 1.0 7 1.5 14 1.5
6
7 8 1.0 9 1.5
8
9 10 1.0 11 1.5
10
11 12 1.0 13 1.5
12
13 14 1.5
14
15 16 1.0 17 1.0 18 1.0 107 1.0
16
17
18
19 20 1.0 21 1.0 22 1.0 108 1.0
20
21
22
23 24 1.0 25 1.0 26 1.0
24
25
26 27 1.5 32 1.5
27 28 1.5 109 1.0
28 29 1.5 51 1.0
29 30 1.0 31 1.5
30
31 32 1.5 34 1.0
32 33 1.0
33
34 35 1.0 39 1.0 43 1.0
35 36 1.0 37 1.0 38 1.0
36
37
38
39 40 1.0 41 1.0 42 1.0
40
41
42
43 44 1.0 45 1.0 46 1.0
44
45
46
47 48 1.0 49 1.0 50 1.0 109 1.0
48
49
50
51 52 1.0 53 1.0 54 1.0
52
53
54 55 1.5 66 1.5
55 56 1.5 110 1.0
56 57 1.5 111 1.0
57 67 1.5 76 1.0
58 59 1.0 60 1.5 67 1.5
59
60 61 1.0 62 1.5
61
62 63 1.0 64 1.5
63
64 65 1.0 66 1.5
65
66 67 1.5
67
68 69 1.0 70 1.0 71 1.0 110 1.0
69
70
71
72 73 1.0 74 1.0 75 1.0 111 1.0
73
74
75
76 77 1.0 78 1.0 79 1.0
77
78
79 80 1.5 85 1.5
80 81 1.5 112 1.0
81 82 1.5 216 1.0
82 83 1.0 84 1.5
83
84 85 1.5 87 1.0
85 86 1.0
86
87 88 1.0 92 1.0 96 1.0
88 89 1.0 90 1.0 91 1.0
89
90
91
92 93 1.0 94 1.0 95 1.0
93
94
95
96 97 1.0 98 1.0 99 1.0
97
98
99
100 101 1.0 102 1.0 103 1.0 112 1.0
101
102
103
104 105 1.0 106 1.0 193 1.0
105
106
107
108
109
110
111
112
113 114 1.5 125 1.5 216 1.0
114 115 1.5 219 1.0
115 116 1.5 220 1.0
116 126 1.5 135 1.0
117 118 1.0 119 1.5 126 1.5

118
119 120 1.0 121 1.5
120
121 122 1.0 123 1.5
122
123 124 1.0 125 1.5
124
125 126 1.5
126
127 128 1.0 129 1.0 130 1.0 219 1.0
128
129
130
131 132 1.0 133 1.0 134 1.0 220 1.0
132
133
134
135 136 1.0 137 1.0 138 1.0
136
137
138 139 1.5 144 1.5
139 140 1.5 221 1.0
140 141 1.5 163 1.0
141 142 1.0 143 1.5
142
143 144 1.5 146 1.0
144 145 1.0
145
146 147 1.0 151 1.0 155 1.0
147 148 1.0 149 1.0 150 1.0
148
149
150
151 152 1.0 153 1.0 154 1.0
152
153
154
155 156 1.0 157 1.0 158 1.0
156
157
158
159 160 1.0 161 1.0 162 1.0 221 1.0
160
161
162
163 164 1.0 165 1.0 166 1.0
164
165
166 167 2.0 178 1.0
167 168 1.0 222 1.0
168 169 2.0 223 1.0
169 179 1.0 188 1.0
170 171 1.0 172 1.5 179 1.5
171
172 173 1.0 174 1.5
173
174 175 1.0 176 1.5
175
176 177 1.0 178 1.5
177
178 179 1.5
179
180 181 1.0 182 1.0 183 1.0 222 1.0
181
182
183
184 185 1.0 186 1.0 187 1.0 223 1.0
185
186
187
188 189 1.0 190 1.0 191 1.0
189
190
191 192 1.5 197 1.5
192 193 1.5 224 1.0
193 194 1.5
194 195 1.0 196 1.5
195
196 197 1.5 199 1.0
197 198 1.0
198
199 200 1.0 204 1.0 208 1.0
200 201 1.0 202 1.0 203 1.0
201
202
203
204 205 1.0 206 1.0 207 1.0
205
206
207
208 209 1.0 210 1.0 211 1.0
209
210
211
212 213 1.0 214 1.0 215 1.0 224 1.0
213
214
215
216 217 1.0 218 1.0
217
218
219
220
221
222
223
224

Crystallographic structure determination of C_4N_4-Me and C_4N_4

The asymmetric unit of the monoclinic crystals of **C_4N_4-Me** (space group C2/c) comprises one half of the molecule and a total of 2.7 disordered co-crystallized dichloromethane molecules, located in positions both inside and outside the macrocycle cavity. The second half of the molecules is generated by rotation around a two-fold crystallographic axis which passes through the centre of the macrocycle. Similarly, the asymmetric unit of the trigonal crystals of **C_4N_4** (space group P-1) also comprises one half of the molecule and a total of 2.2 disordered co-crystallized dichloromethane molecules located inside the macrocycle cavity. In this case, the **C_4N_4** macrocycle is completed by reflection through an inversion centre, which therefore lies at the geometric centre of the cavity. In both cases, the disordered dichloromethane molecules were not included in the final model but were removed with the Platon squeeze tool.[1] Crystallographic data and refinement details are presented in Table S2. Ortep drawings of the **C_4N_4-Me** and **C_4N_4**molecules are shown in **Figure S15**.

[1] Spek, A. L., PLATON SQUEEZE: a tool for the calculation of the disordered solvent contribution to the calculated structure factors, Acta Cryst. 2015, 71, 9-18.

Table S2 - Crystal Data and Details of the Structure Determination for C_4N_4-Me and C_4N_4

(CCDC deposit numbers = 2373754 and 2373755, respectively)

	**C_4N_4-Me**	**C_4N_4**
	**Crystal Data**	
Formula	$C_{100}H_{112}O_{12}$, 2.7(CH_2Cl_2)	$C_{96}H_{104}O_{12}$, 2.2(CH_2Cl_2)
Formula Weight	1735.19	1636.62
Crystal System	monoclinic	Triclinic
Space group	C 2/c (No. 15)	P-1
a, b, c [Å]	37.144(4), 9.674(1), 28.767(3)	9.593(8), 13.908(3), 18.235(5)
α, β, γ [°]	90, 113.448(9), 90	113.342(3), 94.802(11), 97.707(5)
V [$Å^3$]	9483(2)	2188(2)
Z	4	1
ρ (calc) [g/cm^3]	1.215	1.242
μ [mm^{-1}]	0.221	0.206
F(000)	3686	868
	**Data Collection**	
Temperature (K)	100	100
Wavelength [Å]	0.70000	0.70000
θ Min-Max [°]	1.177, 29.740	1.212, 27.789
Dataset hkl range	- 52 : 52; -13 : 13; -38 : 37	- 12 : 12; -18 : 18; -24 : 24
Reflections Total, Unique, R_{int}	83576, 13143, 0.0247	35258, 9957, 0.0599
Observed Data [I > 2.0 σ(I)]	10617	5818
	**Refinement**	
$N_{reflections}$, $N_{parameters}$	13143, 518	9957, 499
R, wR2, S	0.0752, 0.2255, 1.029	0.0642, 0.1744, 1.036
Minimum, Maximum Residual Density [e/$Å^3$]	-0.546, 1.023	-0.249, 0.320

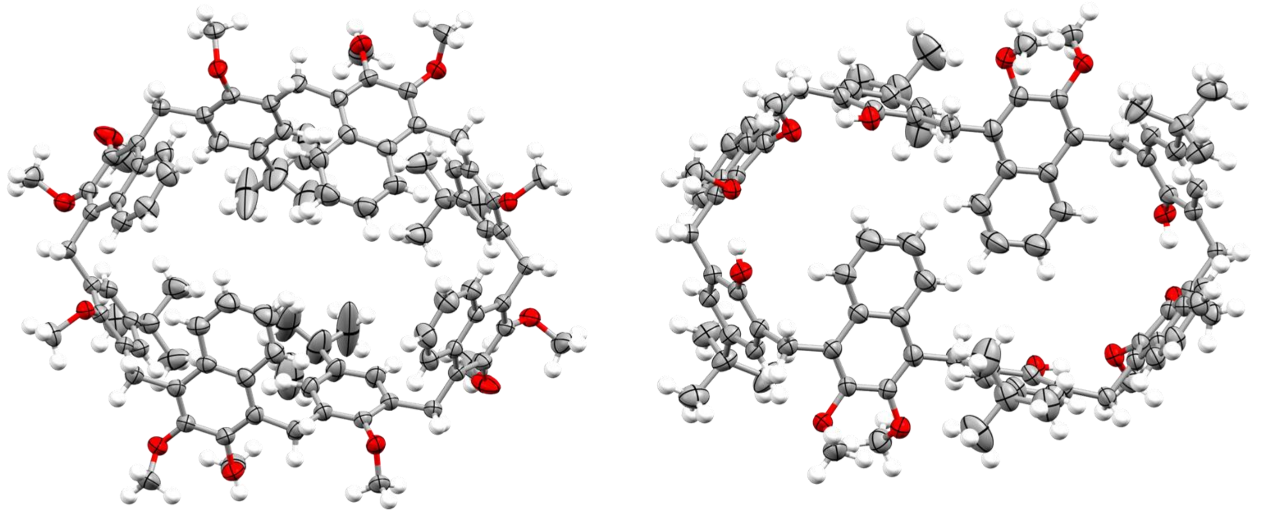

Figure S16: ORTEP drawing of (a) **C_4N_4-Me** and (b) **C_4N_4**. Ellipsoids at 50% probability for the anisotropic thermal factors. Hydrogen atoms refined isotropically are shown in ball-and-stick representation.

Table S3: Dihedral angles, θ, between the mean plane of the bridging methylene groups and the mean planes of the calixarene and naphthalene rings of **C_4N_4-Me** and **C_4N_4**.

	**Dihedral angles[a], θ (°)**							
**Sample**	A	B	C	D	E	F	G	H
**C_4N_4-Me**	64.91	49.74	26.77	72.47	64.91	49.74	26.77	72.47
**C_4N_4**	100.64	99.85	30.21	114.45	100.64	899.85	30,21	114.45

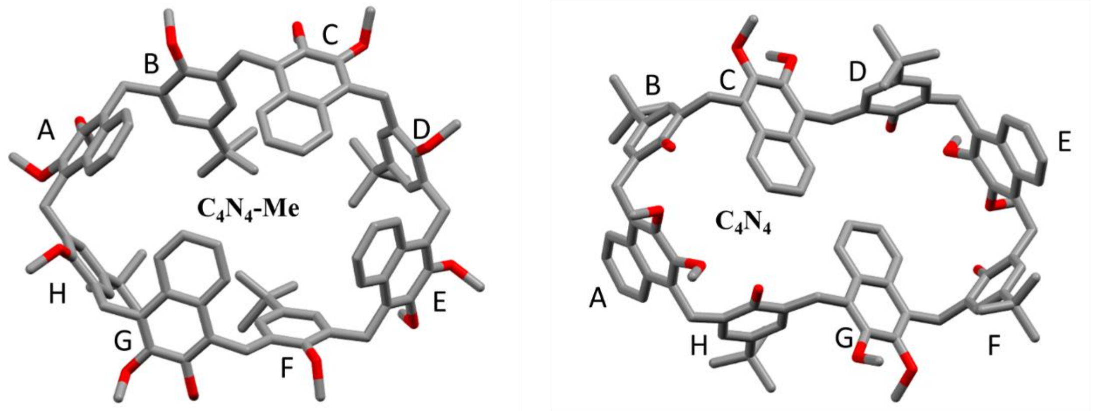

[a] Angles less than 90° indicate that the aromatic ring is tilted inwards towards the centre of the macrocycle, while angles greater than 90 indicate that the aromatic ring is tilted outwards. For **C_4N_4**-Me all angles are tilted inwards, while For C_4N_4 six or the eight rings are tilted outwards.

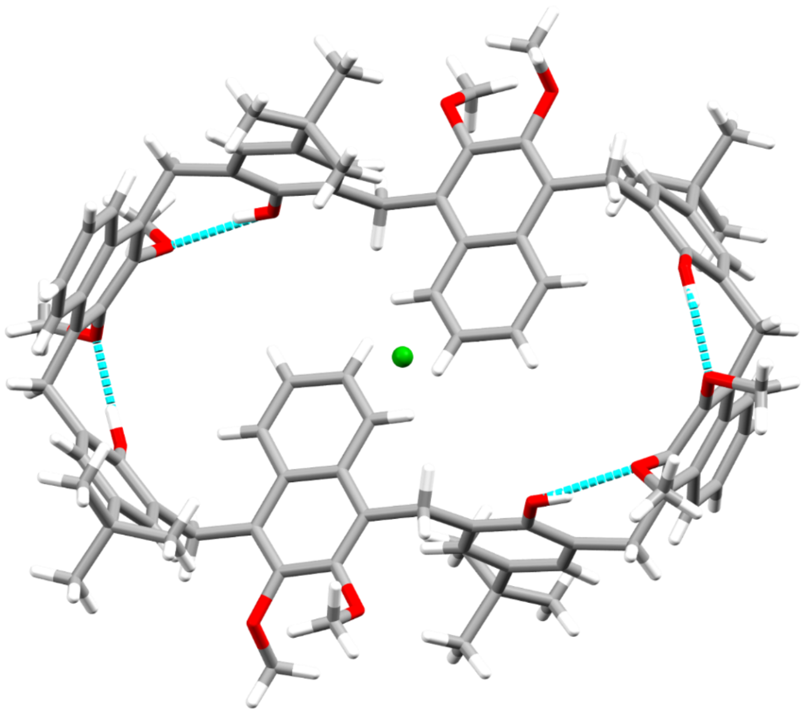

Figure S17: Top view of **C_4N_4-Me**, illustrating the hydrogen bonds between the donor hydroxy groups of the calixarene moieties and the acceptor alkoxy oxygens of the naphthalene moieties. The four hydrogen bonds are composed of two identical pairs, related by inversion through the centre of inversion located at the centre of the macrocycle, illustrated in green. Atoms are shown with CPK colours.

UV-Vis and Fluorescence Characterization C_4N_4-Me

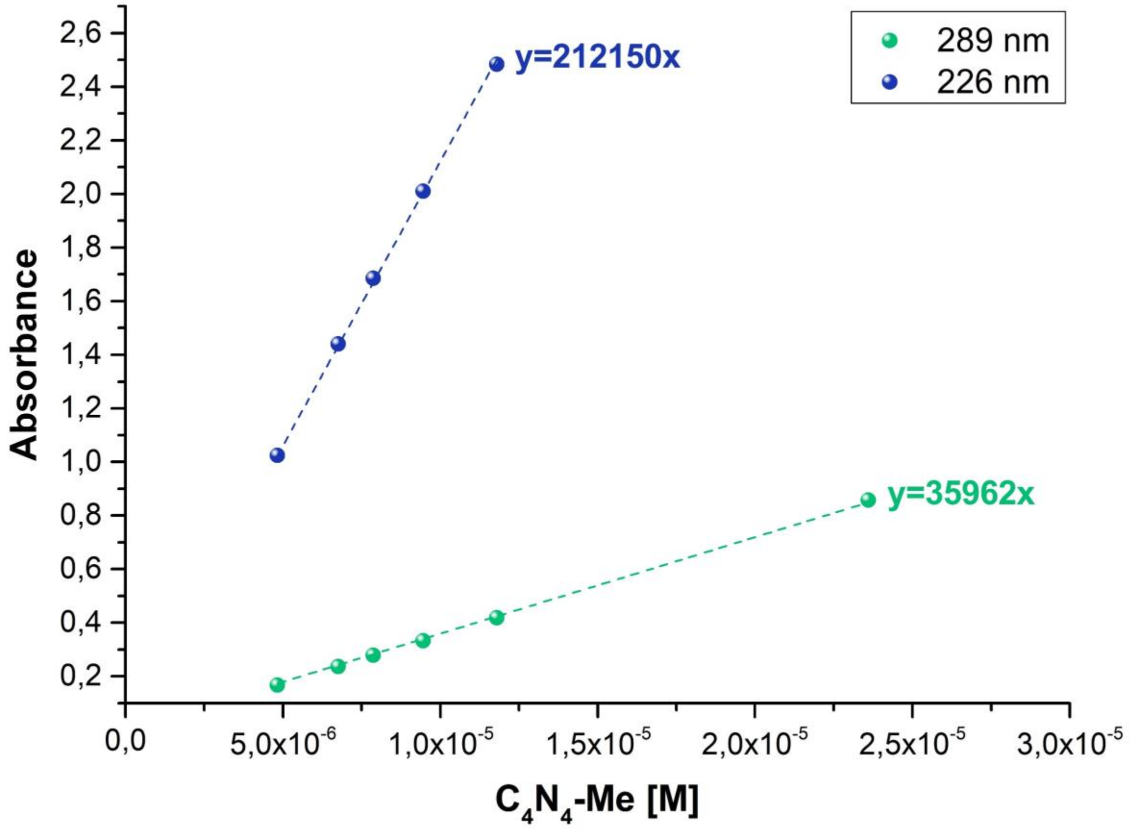

Figure S18: Beer-Lambert plots for the determination of the extinction coefficients of **C_4N_4-Me**.

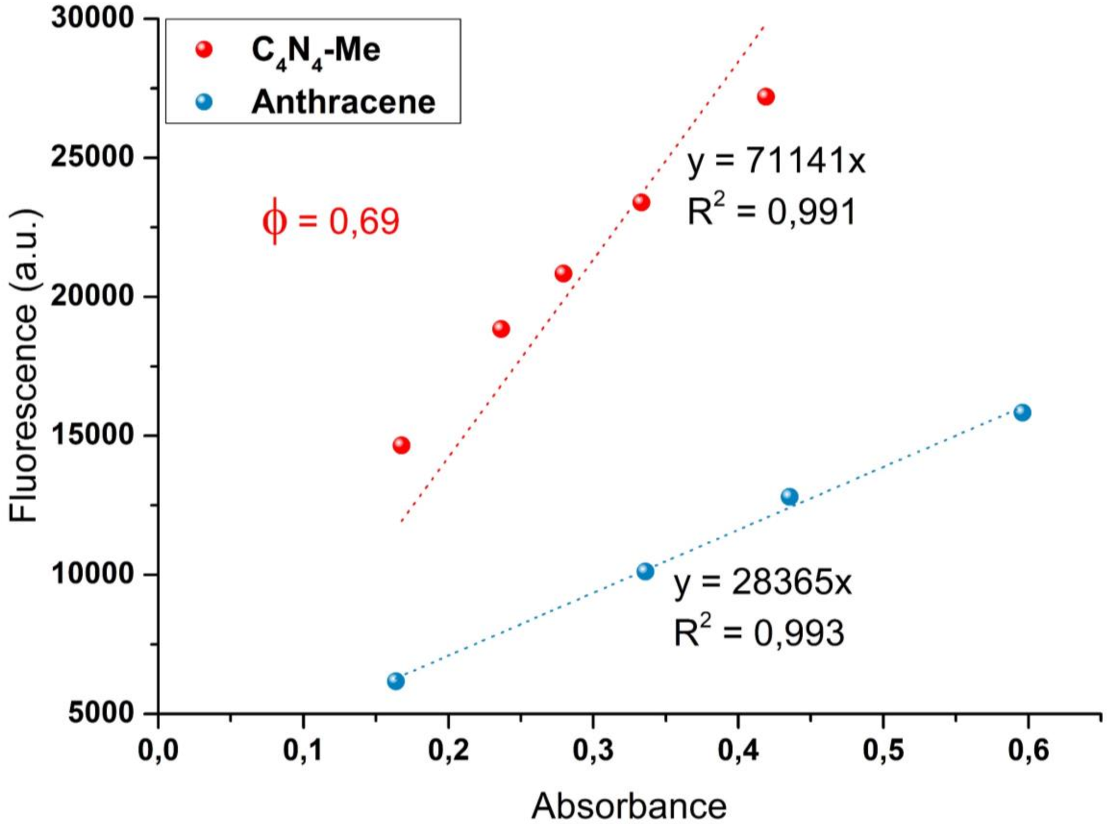

Figure S19: Relative quantum yields for **C_4N_4-Me** (in hexane after excitation at 289 nm) were determined using anthracene (in ethanol) as standards.

Quantitative Distance Determination by NOESY experiment

1,3,5-Alternate (1)

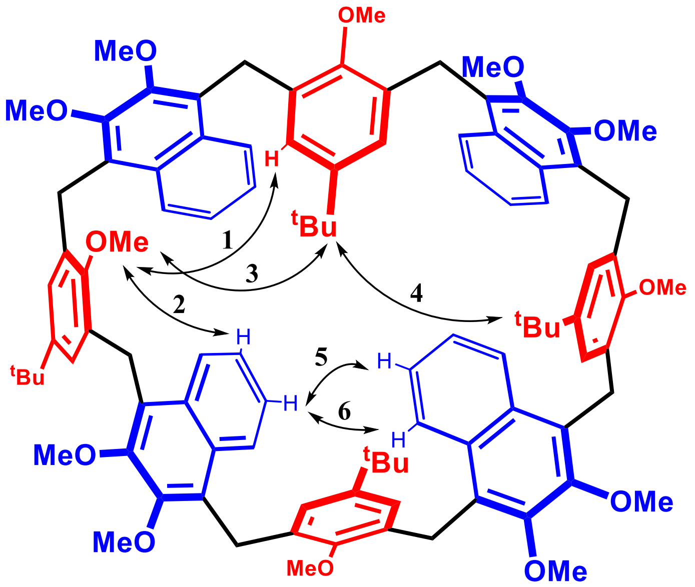

Table S4: Experimental values of interproton distances.

#	Distance (Å)
1	3.61
2	3.53
3	3.30
4	2.61
5	2.55
6	2.87

Figure S20: Chemical drawing of 1,3,5-alternate (I) conformation of **C_4N_4-Me** obtained by molecular dynamics calculation.

1,5-Alternate (1)

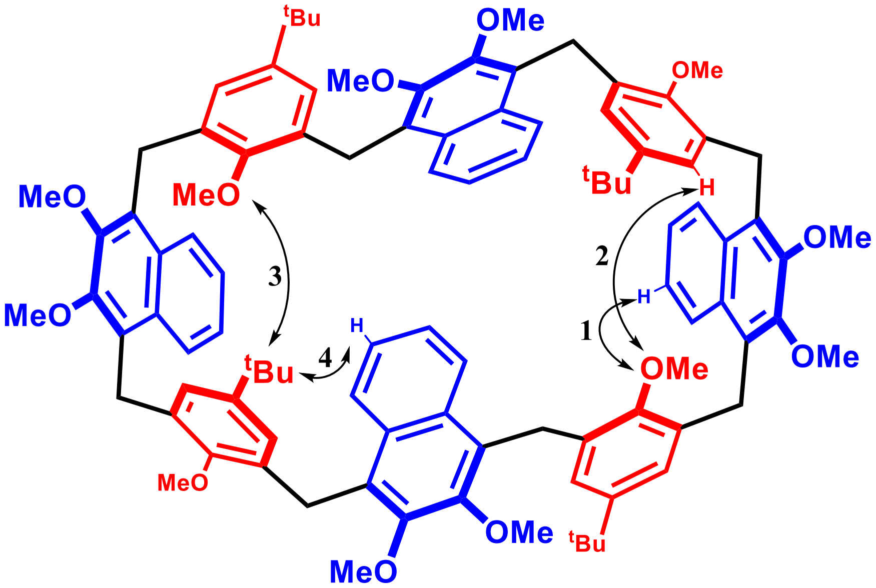

Table S5: Experimental values of interproton distances.

#	Distance (Å)
1	3.62
2	3.89
3	2.71
4	3.60

Figure S21: Chemical drawing of 1, 5-alternate (I) conformation of **C_4N_4-Me** obtained by molecular dynamics calculation.
